# Influence of Deposition Temperature on Cu-BDC Surface-Anchored Metal–Organic Framework Formation

**DOI:** 10.1021/acs.jpcc.4c06638

**Published:** 2024-12-19

**Authors:** Skylar J. Delozier, Dayton L. Maglich, Katherine E. Coffin, Katherine S. Euston, Catherine M. Mauck, Mary E. Anderson

**Affiliations:** † Furman University, Greenville, South Carolina 29613, United States; ‡ Kenyon College, Gambier, Ohio 43022, United States

## Abstract

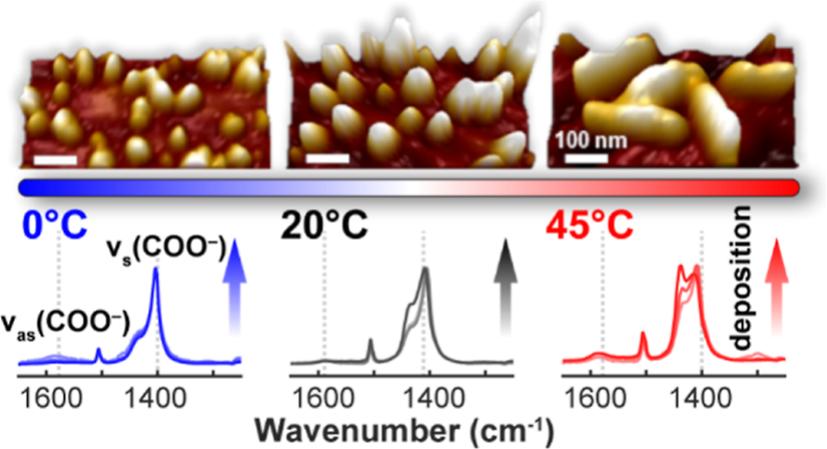

Surface-anchored metal–organic frameworks (surMOFs) are crystalline, nanoporous, supramolecular materials mounted to substrates that have the potential for integration within device architectures relevant for a variety of electronic, photonic, sensing, and gas storage applications. This research investigates the thin film formation of the Cu-BDC (copper benzene-1,4-dicarboxylate) MOF system on a carboxylic acid-terminated self-assembled monolayer by alternating deposition of solution-phase inorganic and organic precursors. X-ray diffraction (XRD) and atomic force microscopy (AFM) characterization demonstrate that crystalline Cu-BDC thin films are formed via Volmer–Weber growth. Changes in film morphology as the deposition temperature increases are seen by AFM with more isolated nanorod-like crystallites observed at lower temperatures, while vertical nanoplatelet-like structures form at higher temperatures. At 45 °C, the nanoplatelets are observed to be composed of fused nanorod segments aligned in one direction. Ellipsometry confirms that both increasing temperature and number of deposition cycles yield more film deposition in agreement with infrared reflectance–absorbance spectroscopy (IRRAS) that was further used to characterize the chemical binding and orientation in the surface-bound Cu-BDC nanostructures. In addition to observing strong preferred orientation of the nanostructures from the enhancement of the symmetric carboxylate stretch and absence of the antisymmetric stretch, IRRAS results show the emergence of a new binding motif associated with segmented nanoplatelet formation at a higher deposition temperature as well as at high surface coverage after 12 deposition cycles. From the appearance of this higher frequency symmetric carboxylate peak alongside peak splitting in BDC deformation modes, IRRAS data support the presence of a strained configuration that accompanies the appearance of Cu-BDC segmented nanoplatelets observed by AFM.

## Introduction

Solution-phase assembly of metal–organic frameworks (MOFs), which are a class of crystalline, nanoporous, supramolecular materials composed of inorganic nodes and organic linkers, has produced a vast number of structures and compositions with potential to impact a wide range of fields from medicine to sustainable energy.^1−10^ One approach toward the integration of this nanoporous material into hierarchical architectures is the solution-phase layer-by-layer deposition of organic and inorganic precursors to form surface-anchored MOFs (surMOFs). These surMOFs are typically anchored to substrates via self-assembled monolayers (SAMs) and are often studied as ultrathin films with sub-100 nm thicknesses.^11−18^ Key variables to tune the interfacial interactions that direct the bottom-up assembly and impact the resulting structure of the surMOF thin films are surface functionalization,^18−23^ deposition method (e.g., immersion vs spray),^23−26^ and solution temperature.^18,27^ In combination with atomic force and scanning electron microscopy (AFM and SEM, respectively) characterization, surMOF films are commonly characterized by ensemble techniques such as ellipsometry, quartz crystal microbalance (QCM), surface plasmon resonance (SPR), X-ray diffraction (XRD), and IR spectroscopy. Microscopy investigates film morphology on the nanoscale; ellipsometry, QCM, and SPR determine the average amount of film material deposited; and XRD probes crystallinity. IR spectroscopy investigates the chemical bonding within the MOF structure and can investigate the preferred orientation of MOF structures when polarized techniques such as infrared reflectance–absorbance spectroscopy (IRRAS) are used.

This study focuses on the impact of deposition temperature on the thin film formation of the copper-paddlewheel MOF system Cu-BDC (Cu(II) benzene-1,4-dicarboxylate) with characterization by AFM, ellipsometry, XRD, and IRRAS. Cu-BDC contains a paddlewheel of copper(II) dimers and is also referred to as Cu-MOF-2 because it is an analogue of MOF-2, which contains a paddlewheel of zinc(II) dimers. The central unit of the metal node for Cu-BDC is a dimer of copper(II) ions coordinated in the equatorial plane by four carboxylate groups from the benzene 1,4-dicarboxylate (BDC) linkers and is occupied postsynthesis in the axial positions by water or solvent molecules that can be removed upon subsequent activation, which is typically heating under vacuum.^28−31^ As a powder, Cu-BDC has *C*_2_ symmetry;^32,33^ however, previous research regarding the system as a surMOF has shown that a high-symmetry *P*_4_ structure forms.^34,35^ Applications for Cu-BDC range from catalysis and sensing technologies to their use as antimicrobial coatings.^33,36−38^

Recently, Cu-BDC surMOF thin film formation by alternating, sequential, solution-phase immersion deposition on carboxyl- and hydroxyl-terminated SAMs was investigated with AFM characterization revealing a Volmer–Weber growth mechanism in which the thin films exhibited significant morphological differences.^23^ Standing-up (vertical) nanorods were found anchored to the carboxyl-terminated SAM, whereas lying-down (horizontal) nanorods were formed on the hydroxyl-terminated SAM. XRD characterization confirmed crystallinity and indicated a difference in preferred orientation for the vertical versus horizontal nanorods. These findings were consistent with research studies for other systems, such as HKUST-1 and Cu_2_BDC_2_DABCO MOF systems,^18−21^ on which different crystal orientations were observed based on the underlying chemical functionality. Additionally, in a recent research study from Dhanapala et al., it was found that different surface functionalities did not impact the film morphology when analogous spray deposition methods for the deposition of Cu-BDC thin films were used.^23^ This difference is postulated to be due to the formation of a kinetic product for the spray deposition, whereas the immersion method produces a thermodynamic product.

For other MOF systems, the impact of temperature on the nanostructure of surMOF films has been previously shown to impact the morphology and crystal orientation. The combination of microscopy techniques with XRD and IRRAS reveals information about the crystallite shape and internal structure. One key demonstration of this was by Terfort and co-workers investigating the Cu_2_BDC_2_DABCO MOF structure that contains a copper-paddle wheel coordinated at all 6 sites (axial and equatorial), which differs from the Cu-BDC herein that only has organic linkers coordinated at the equatorial paddlewheel locations.^18^ Alongside the temperature (5–60 °C), they investigated the impact of different SAM surface functional groups (amine and carboxylic acid) on surMOF formation. A significant difference in morphology was seen for the different functional groups, with horizontal platelets observed by SEM for the amine and vertical platelets observed for the carboxylic acid. This difference in orientation was supported by IRRAS showing a substantial difference in the appearance of the asymmetric versus symmetric carboxylate stretch of the MOF and by XRD revealing different relative intensities between the 100 and 001 lattice planes. For this system, increased temperature reduced the amount of preferred orientation with a mixture of horizontal and vertical platelets observed by microscopy and reflected in the corresponding IRRAS and XRD data.^18^

A later study investigated alternative organic linkers, replacing BDC, to see how the acid dissociation properties (p*K*_a_) of the dicarboxylate linker induced a preferred orientation on amine SAMs. These alternative linkers also resulted in subtle shifts in the frequency of the IRRAS vibrations, particularly notable for asymmetric COO^–^ but also for symmetric COO^–^.^39^ The separation value (Δ) between the antisymmetric and symmetric carboxylate stretch in MOFs containing BDC linkers is usually taken as a measure of the metal binding mode, where the magnitude of Δ is thought to increase as binding goes from chelating to bridging to monodentate.^40−42^ One recent study used values of Δ to distinguish between different layered structures that could be chemically generated from bulk Cu-BDC.^43^ These and other studies have demonstrated that IRRAS is a powerful technique to analyze the chemical binding in Cu-BDC surMOFs.

At lower, ambient, and elevated temperatures (0, 20, and 45 °C) and as a function of deposition cycle (4, 8, and 12 L), this study investigates the thin film formation of the Cu-BDC surMOF system on carboxylic acid-terminated SAMs on gold substrates. These surMOF thin films underwent characterization by ellipsometry every 4 deposition cycles to determine the optical thickness of the film. After the sample had undergone the target number of deposition cycles, samples were analyzed using AFM, XRD, and IRRAS to observe film morphology and roughness, confirm the presence of crystalline material, and investigate chemical binding as well as the preferred orientation within the structure. Analysis of these findings reveals unique morphological features observed by AFM, which are characterized by the appearance of shifted vibrational modes associated with the Cu-BDC binding and pore geometry. This provides nano- and molecular-scale insights into the impact of temperature on the nanocrystallite assembled throughout the solution-phase deposition process to compose the Cu-BDC surMOF thin film.

## Experimental Section

### Materials

SurMOF formation incorporated 16-mercaptohexadecanoic acid [MHDA] (90%), copper(II) acetate monohydrate (≥98%), and benzene-1,4-dicarboxylic acid [H_2_BDC] (98%), all purchased from Sigma-Aldrich. Ethanol (200 proof ACS/USP grade) was acquired from Pharmco by Greenfield Global. Gold-coated (100 nm) silicon wafers with an adhesion titanium layer (5 nm) were obtained from Platypus Technologies. All chemicals were used as received.

### Preparation of Layer-by-Layer (LbL) Cu-BDC surMOFs

In a similar manner to previous studies,^23,27,44^ Cu-BDC thin films were assembled via a solution-phase layer-by-layer (LbL) deposition method onto carboxylic acid-terminated SAMs on gold substrates. Gold substrates were immersed in a 1 mM ethanolic solution of 16-mercaptohexadecanoic acid for a duration of 1 h before being rinsed with ethanol and dried under a flow of N_2_. The Cu-BDC MOF system was deposited using an alternating, sequential, LbL deposition method with ethanolic solutions of 1 mM copper(II) acetate monohydrate as the metal precursor and 0.1 mM H_2_BDC as the organic precursor. MHDA-functionalized substrates were submerged sequentially in these metal and organic precursors for 5 and 10 min, respectively, with ethanol rinsing and N_2_ drying between each deposition. For deposition above and below room temperature, precursor solutions were either heated to 45 °C or cooled to 0 °C prior to submerging the substrates. Deposition in the metal and organic precursors consecutively comprises one deposition cycle, or surMOF thin-film layer (L). Samples were stored in a desiccator following the MOF assembly and characterized by ellipsometry, AFM, XRD, and IRRAS to determine the film thickness, morphology, crystallinity, and chemical bonding, respectively.

### Ellipsometry

Optical film thickness data was routinely gathered before and after SAM formation as well as after every four deposition cycles using a single-wavelength, fixed-angle LSE Stokes ellipsometer (Gaertner Scientific Corporation). Measurements were conducted by using a helium–neon laser with a wavelength of 6328 Å at a 70° incidence angle. To determine the optical film thickness, the Gaertner ellipsometer measurement program (LGEMP) was used to fit the measured psi and delta data obtained for the films. The model included measured values for each gold substrate’s index of refraction (*n*_s_) and extinction coefficient (*k*_s_) alongside set values for the film’s index of refraction (*n*_f_) at 1.5 and extinction coefficient (*k*_f_) value at 0.^23,45^ A minimum of 6 spots were collected per sample. Average optical thickness and corresponding standard deviation presented are representative of at least four replicates.

### Atomic Force Microscopy (AFM)

Surface morphology was imaged using a Park Systems NX10 AFM with a PPP-NCH 10 M probe (42 N/m force constant) in noncontact mode. Images with dimensions of 2.5 μm × 2.5 μm (256 × 256 pixels) were taken using an XY single-module flexure, closed control scanner with a scan range of 50 μm × 50 μm. Data collection utilized the SmartScan adaptive operation mode for the NX10 with a default setting for scan rate and set point of 1 Hz/line and 12 nm, respectively; however, due to the roughness of the surfaces imaged by AFM in this study, typical adaptive scan rates and set points were ∼0.25 Hz/line and ∼10 nm, respectively. Surface roughness (*R*_q_) and morphology were studied by using XEI data processing and analysis software (Park Systems). Average roughness values and associated standard deviations were calculated for all deposition conditions (temperature and number of layers) from a minimum of four samples with at least three images each.

### Powder X-ray Diffraction (XRD)

XRD patterns for Cu-BDC thin films were collected at room temperature by using a Rigaku Miniflex II benchtop diffractometer with settings of 30 kV and 15 mA with Cu K_α_ radiation (λ = 1.5418 Å). A sampling width of 0.03° with a scan speed of 1.00° per minute was used to collect diffraction patterns from the 7 to 18° 2θ range. Data was collected for a minimum of four samples per deposition condition.

### Infrared Reflectance–Absorbance Spectroscopy (IRRAS)

Thin film samples on Au were analyzed with IRRAS under vacuum using a Bruker Vertex 80v FTIR spectrometer coupled to a liquid-nitrogen-cooled MCT detector. Samples were mounted for *p*-polarized reflectance measurements in a Harrick AutoSeagull variable angle reflectance accessory with the incident angle set to 82° and a KRS-5 wire grid polarizer. The background was acquired on a gold substrate under vacuum. Spectra were converted to absorbance and then baseline-corrected in Bruker OPUS (8.4) and Origin Lab software.

## Results and Discussion

Thin films composed of Cu-BDC surMOFs anchored to gold substrates by carboxylic-acid-terminated SAMs were deposited at lowered, ambient, and elevated temperatures (0, 20, and 45 °C) to investigate the impact of the temperature on the film structure. Films were fabricated with different numbers of deposition cycles (4, 8, and 12 L) to investigate how these structures may evolve as more material is deposited. SurMOF thin film characterization was routinely conducted via ellipsometry every 4 deposition cycles to determine the optical thickness of the film. Upon completion of the desired number of deposition cycles, samples were analyzed by using atomic force microscopy (AFM), X-ray diffractometry (XRD), and infrared reflectance–absorbance spectroscopy (IRRAS) to image the surface morphology, verify the crystallinity, and examine the molecular structure as well as orientation associated with organic linker vibrations.

### Structural Characterization by AFM, Ellipsometry, and XRD

**Figure 1:**
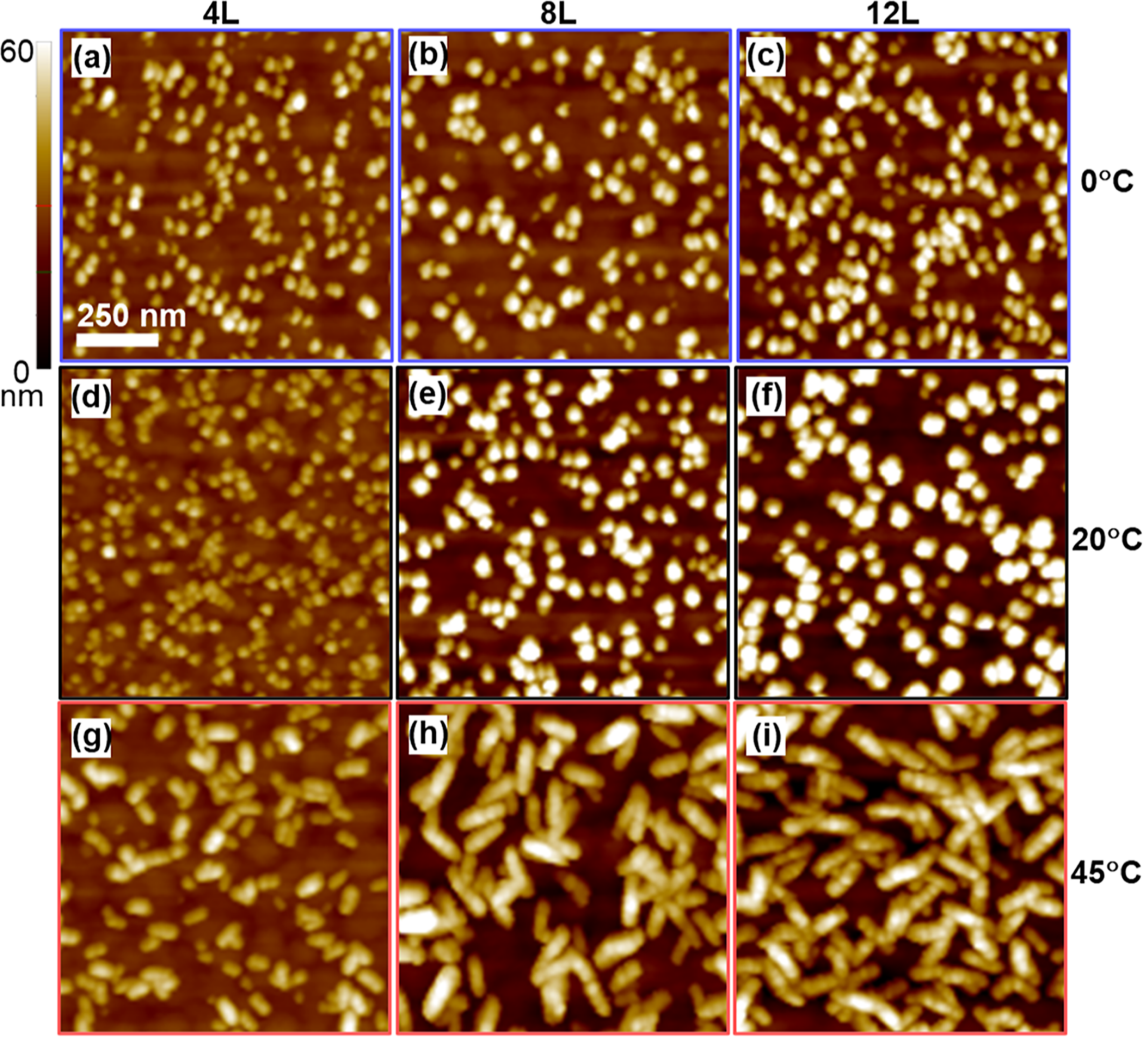
Representative AFM images (1 μm × 1 μm) of Cu-BDC on carboxylic acid-functionalized substrates after 4, 8, and 12 deposition cycles (L). These images are regions extracted from 2.5 μm × 2.5 μm images to highlight the tall, nanorod structures observed for samples created at (a–c) lower and (d–f) ambient temperatures in contrast to the segmented platelets of fused nanorods seen for samples fabricated at (g–i) elevated temperatures.

AFM images of Cu-BDC surMOF thin films deposited at lowered, ambient, and elevated temperatures after 4, 8, and 12 deposition cycles on MHDA-functionalized gold substrates are shown in Figure 1. These images suggest that a Volmer–Weber growth mechanism is observed for all temperatures consistent with previous research findings for this and other related systems.^23,27^ For thin films deposited at lower temperatures (Figure 1a–c), isolated circular nanocrystallites are observed with the z-height increasing with more deposition cycles. SurMOFs deposited at ambient temperatures (Figure 1d–f) had similarly shaped particles with a greater height change across deposition cycles. Additionally, the density (number of particles per area) of these particles is increased relative to the films formed at lower temperatures. These structures are in accord with those previously reported for these films fabricated at room temperature, which were assigned to be nanorods growing vertically relative to the substrate.^23^ At elevated temperatures (Figure 1g–i), the deposited Cu-BDC surMOF appears to have an elongated, bumpy, vertical, nanoplatelet-like structure, with each bump postulated to be a nanorod segment. As seen for the lower temperatures, the height of these features increases with more deposition cycles; but unlike at the other temperatures, the length of these features increases significantly between four and eight deposition cycles. Additionally, segmented features along the elongated structure become readily apparent after eight deposition cycles (Figure 1h) with 3–5 segments, which were only visible in a few of the elongated structures after four deposition cycles (Figure 1g) with 2–3 segments. This segmentation of the nanostructure features is prominent in appearance after 12 cycles (Figure 1i).

AFM images in Figure 1 show 1 μm × 1 μm regions of the samples to permit the clear viewing of nanoscale features, such as the segmented nature of the features observed in Figure 1h,i. These are representative regions selected from larger 2.5 × 2.5 μm images, which are provided in Figure S1 to show sample homogeneity. To show the consistency of cropped images with higher-resolution images, Figure S2 contains a representative image captured with 1 μm × 1 μm dimensions.

**Figure 2:**
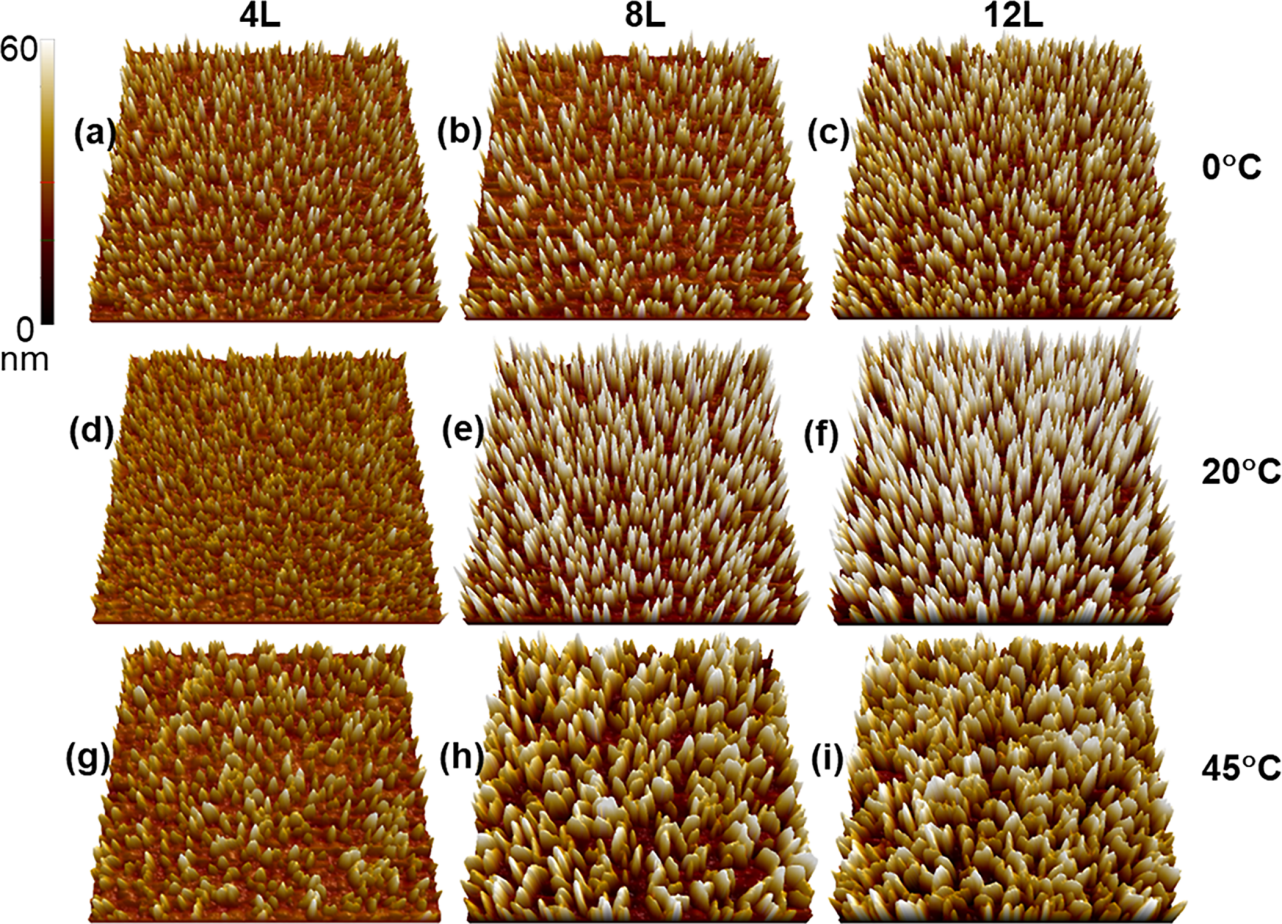
Three-dimensional images (2.5 μm × 2.5 μm) of Cu-BDC surMOFs on carboxylic acid-functionalized substrates. These perspectives were rendered from AFM images to convey the structure and density of the nucleated crystals formed throughout the deposition process after 4, 8, and 12 deposition cycles (L) at varying precursor temperatures. These images represent samples with deposition at (a–c) lower, (d–f) ambient, and (g–i) elevated temperatures.

Additionally, three-dimensional renderings of these images are provided in Figures 2 and S3 to assist in visualization of the morphological features found by AFM. Figure 2 conveys the shape similarities alongside the height and density differences observed for the Cu-BDC surMOF films deposited at lower and ambient temperatures (Figure 2a–c,d–f). The isolated crystallites observed at these temperatures are consistent with nanorods protruding vertically from the substrate. As all images in Figure 2 are set to the same z-scale, an increase in height with increasing number of deposition cycles is apparent alongside the observation that the density of the particles formed at ambient temperatures (Figure 2d–f) is greater than that found for the films created at lower temperatures (Figure 2a–c). To further convey the differences in heights observed for these samples, representative line scans are included in Figure S4. After 12 deposition cycles, the maximum particle heights observed were 45 and 75 nm for lower and ambient temperatures, respectively.

The unique elongated structures observed for the Cu-BDC surMOF films formed at elevated temperatures are further investigated by the three-dimensional visualization in Figure 2g–i and compared to those formed at lower and ambient temperatures (Figure 2a–c,d–f). These elongated nanoparticles, whose segmentation is evident in Figure 1g–i, are revealed in Figure 2g–i as vertical nanoplatelets that form at an elevated temperature. Previous research investigated the room-temperature film formation of Cu-BDC on hydroxyl-terminated SAMs and also observed that elongated particles were formed on the substrate.^23^ In contrast to the present study, the nanostructures found on those hydroxyl-terminated SAMs had smooth curvature in their features’ morphology and were described as horizontal, lying-down rods. The features of samples in the present study created at elevated temperatures on carboxylic-acid terminated SAMs are elongated particles, but are dissimilar from that horizontal rod structure observed at ambient temperature on a different surface functional group. Instead, the segments of the elongated particles seen in this study (Figures 1g–i and 2g–i) are quite similar to the isolated nanorods seen at the lower and ambient temperatures, and thus, the particles deposited at an elevated temperature are described as nanoplatelets composed of fused vertical nanorod segments. Close inspection of Figure 2h,i supports this because the top surfaces of the nanoplatelets appear as discontinuous ridgelines with each peak an individual nanorod segment. The representative line scans (Figure S4g–i) further illustrate this and highlight the difference in the platelet morphology from the nanorod morphology observed at lower temperatures (Figure S4a–f). Platelet structures were previously observed at elevated temperatures in a different copper-paddlewheel MOF structure, Cu_2_BDC_2_DABCO.^18^

As the alignment of nanorods into platelets has occurred at elevated temperatures, it is of note that some regions within the lower and ambient images (Figure 1a–i) seem to have particles that spatially align themselves side by side in one direction. One would anticipate that more interaction between the nanorods would occur with increasing deposition and density of Cu-BDC nanorod crystallites. The height of the platelet structures in Figure 2g–i increases with more deposition cycles. It is noteworthy that the features for the film formed by 12 deposition cycles at the elevated temperature are similar in height to those observed for the film formed with the same number of cycles at ambient temperatures. Additionally, it is interesting to see that the nanoplatelets have different orientations that are not aligned with one another over long distances, suggesting that this directionality is likely not influenced by deposition conditions, such as rinsing or the vertical orientation maintained during the deposition process.

**Figure 3:**
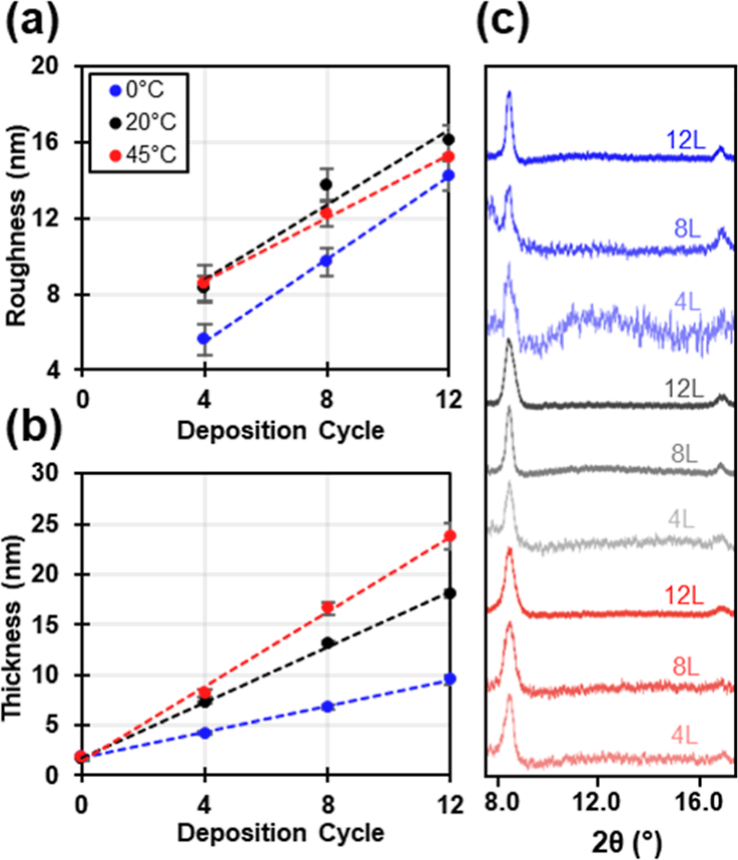
Cu-BDC surMOF samples fabricated at 0, 20, and 45 °C after 4, 8, and 12 deposition cycles (L). (a) Average roughness values and standard deviations from AFM images (2.5 μm × 2.5 μm) and (b) average ellipsometric film thicknesses with standard deviations are shown. (c) Representative XRD patterns collected for films fabricated with different conditions.

To thoroughly and quantitatively investigate these film morphologies, multiple samples and replicates were investigated by AFM. This compilation of data for the average roughness (*R*_q_) at the different temperatures as a function of the deposition cycle is shown in Figure 3a. While AFM showed that the lower and ambient temperatures had similar morphologies (Figure 1a–f), the roughness is significantly higher for the films deposited at ambient temperatures. This is congruent with the height and density differences observed for these two sample sets fabricated at different temperatures (Figure 2a–f) and is also in agreement with less material being deposited for the sample fabricated at lower temperatures. Ellipsometry data (Figures 3b and S5) show that the amount of film deposited per layer at ambient temperatures compared to lower temperatures was 1.4 and 0.64 nm per deposition cycle, respectively. Despite different morphological features of nanorods for ambient and nanoplatelets for elevated temperature deposition, AFM roughness data for these samples are within standard deviations of one another (Figure 3a). While the roughness is the same, the film thickness is higher at elevated temperatures. Thicknesses are measured by ellipsometry to be 1.9 nm deposited per cycle at elevated temperatures (Figure S5). For all samples, a general increase in film roughness and thickness occurred with an increase in the number of deposition cycles (Figure 3a,b). Higher deposition temperatures were associated with increased film deposition observed by ellipsometry (Figure 3b). It is postulated that the increased diffusion occurring at higher temperatures results in more film nucleation and growth because there is an increased probability of interaction between the precursor components in solution and the substrate. XRD patterns for all samples support the deposition of crystalline material, with a decrease in the signal-to-noise ratio observed as the number of deposition cycles increases, which is consistent with an increased amount of crystalline material on the substrate (Figure 3c).

### Spectroscopic Characterization by IRRAS

**Figure 4:**
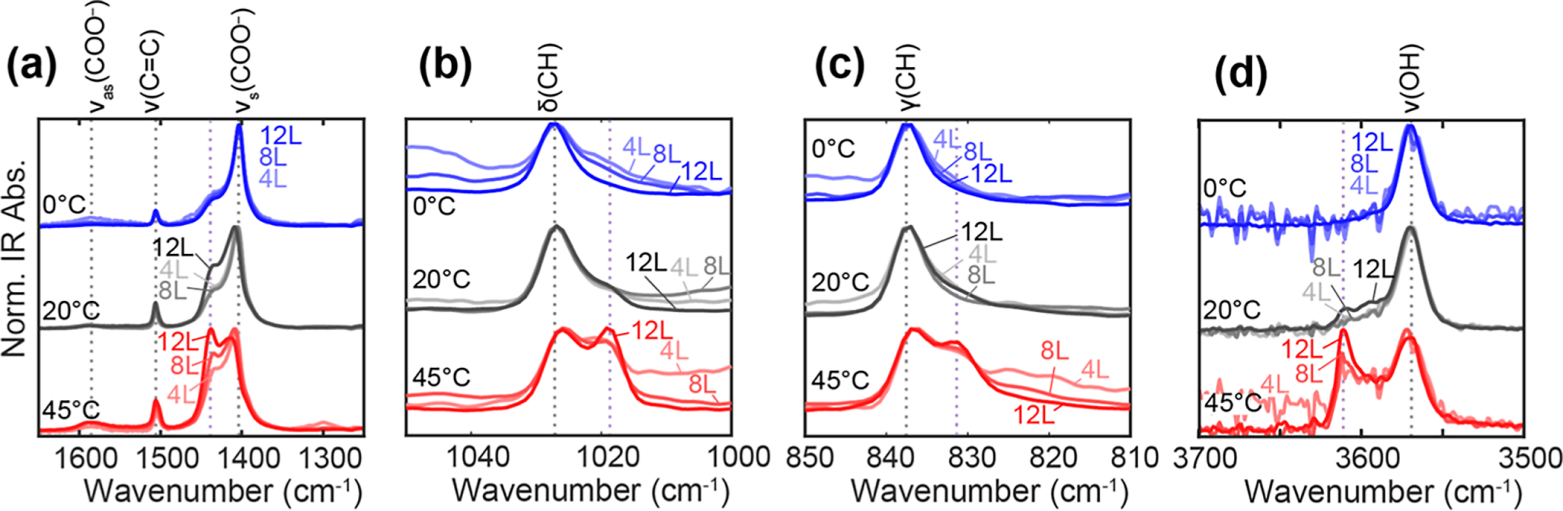
Normalized IRRA spectra of films deposited at 0 °C (blue), room temperature (black), and 45 °C (red) for 4, 8, and 12 deposition cycles (L) for the (a) carboxylate and ring stretching region; (b,c) CH deformation modes δ(CH) and γ(CH); and (d) hydroxyl stretching region.

For Cu-BDC surMOF films, the IRRA spectra for the carboxylate, C–H deformations, and hydroxyl vibrations are given in Figure 4a–d. The spectra are normalized to facilitate a comparison of the regions that show significant changes with increased deposition cycles and temperature. Without normalization, the most prominent peak is the symmetric carboxylate stretch ν_s_(COO^–^), which appears between 1403 and 1414 cm^–1^. The antisymmetric carboxylate stretch ν_as_(COO^–^) appears at 1580–1590 cm^–1^, but has very low intensity in the IRRAS data. The sharp, medium-intensity BDC ring stretch ν(C=C) is observed at 1507 cm^–1^, assigned to the ν_19b_ mode of Wilson’s notation.^46^ C–H deformation bands at 829 and 1015 cm^–1^ are assigned to γ(CH)_11_ and δ(CH)_18a_, respectively.^47^ A sharp peak at 3570 cm^–1^ is assigned to a ν(OH) hydroxyl stretch.^23,48,49^ The methylene stretching frequencies of the MHDA SAM are visible at 2920 and 2850 cm^–1^.^50^ The dominant vibrational features for Cu-BDC surMOF films come from the BDC linker, with the full spectra for each deposition temperature and cycle shown in Figure S6.

In agreement with the ellipsometry measurements, the peak intensity generally increases as the film thickness grows and the signal-to-noise ratio is best for the highest number of deposition cycles (12 L) at each temperature. This can be seen clearly in the ν(OH) peak when normalizing the IRRAS data to the MHDA signal, whose intensity should be constant among all samples (Figure S7). To determine the impact of increasing film deposition cycles on the IR signal, the integrated peak areas were computed for band regions corresponding to ν_s_(COO^–^), ν(C=C), ν_as_(COO^–^), and ν(OH). Those areas are plotted as a function of the deposition cycle for all temperatures in Figure S8 and provided in Table 1. While a relatively monotonic increase in peak area is observed from 4 to 12 deposition cycles at the lowered temperature (0 °C), there is a deviation in the peak area trends as deposition temperature increases, with the highest areas at 45 °C deposition seen in the 8 L samples. If the morphology of the Cu-BDC surMOF structure remained the same at increasing deposition temperature, linear growth in intensity would be expected as in the ellipsometry data. Yet, because IRRAS is sensitive to the chemical binding environment of the BDC linker, the observed deviation from linearity suggests the appearance of a different structural motif that leads to additional growth in-plane instead of out-of-plane, which becomes accessible at higher temperatures.

**Table 1:** Peak Position (cm^–1^), Separation Value (Δ), Integrated Area, and Orientation Factor for Select Vibrational Modes

sample	LbL	LbL	LbL	LbL	LbL	LbL	LbL	LbL	LbL	spray	powder
*T* (°C)	0	0	0	20	20	20	45	45	45	20	
L (cycle)	4	8	12	4	8	12	4	8	12	12	

mode	Frequency (cm^–1^)										
ν_as_(COO^–^)	1586.2	1589.1	1581.3	1590.0	1586.2	1590.0	1585.2	1588.1	1586.2	1577.5	1605
ν(C=C)	1506.1	1507.1	1507.1	1507.1	1507.1	1507.1	1506.1	1506.1	1507.1	1508.1	1507
ν_s_(COO^–^)_1_^a^	1403.6	1403.8	1404.1	1407.0	1405.5	1410.2	1408.2	1410.9	1414.6	1410.7	1391
ν_s_(COO^–^)_2_^a^	1430.2	1429.3	1428.1	1429.6	1431.3	1435.1	1432.9	1435.6	1438.8	1440.6	

Δ values											
Δν(COO^–^)_1_^b^	183	185	177	183	181	180	177	177	172	167	214
Δν(COO^–^)_2_^b^	156	160	153	160	155	155	152	153	147	137	

mode	Integrated Area										
ν_as_(COO^–^)	0.21	0.16	0.13	0.09	0.30	0.19	0.20	0.77	0.65	1.52	13.6
ν(C=C)	0.10	0.17	0.28	0.11	0.42	0.51	0.19	0.64	0.62	0.30	
ν_s_(COO^–^)	3.28	5.03	8.38	3.42	12.60	12.86	5.06	14.77	13.09	3.32	18.1

orientation	Ratio										
*F*^c^	0.94	0.97	0.99	0.97	0.98	0.99	0.96	0.95	0.95	0.69	0.57

a Peak centers from Lorentzian peak fits, where ν_1_ is the lower frequency peak and ν_2_ is the higher frequency peak.

b Separation value of asymmetric and symmetric stretches of carboxylate groups indicating mode of binding.

c Orientation factor *F* = *A*_s_/(*A*_s_ + *A*_vs_) where *A*_s_ and *A*_as_ are integrated areas of ν_s_(COO̅^-^) and ν_as_(COO^-^), respectively.

Notably, the antisymmetric carboxylate stretch ν_as_(COO^–^) at 1580–1590 cm^–1^ is almost absent in the IRRA spectra in Figure 4a due to the anisotropic growth of Cu-BDC surMOF. Because the symmetric and antisymmetric carboxylate stretches in the BDC linker have orthogonal transition dipole moments, their intensity in IRRAS can be used to determine the oriented growth of the surMOF relative to the substrate.^18,51^ In the IRRAS measurement, *p*-polarized light will interact strongly with vibrations whose transition dipoles are aligned normal to the gold substrate, enhancing their intensity.^52^ In contrast, transition dipole components that are parallel to the substrate will be silent in IRRAS. Therefore, in Figure 4a, the intensity of ν_as_(COO^–^) is suppressed at all temperatures and deposition cycles, while that of ν_s_(COO^–^) is enhanced. Cu-BDC film orientation can be estimated using the integrated areas of the symmetric and antisymmetric carboxylate stretches (*A*_s_ and *A*_as_) to arrive at the anisotropy ratio, or orientation factor *F*, where *F* = *A*_s_/(*A*_s_ + *A*_as_).^51^ The peak positions and areas for the carboxylate peaks as well as ν(C=C) are summarized in Table 1, along with orientation factor *F* for each film. The IRRAS results confirm highly oriented growth in which ν_s_(COO^–^) has preferred alignment perpendicular to the substrate, with *F* = 0.94–0.99 in all surMOF films, which corresponds to the Cu–Cu axis of the Cu-BDC paddlewheel orienting parallel to the substrate in the surMOF film. The anisotropic order evident by IRRAS for these Cu-BDC surMOF films is further supported by comparing 12 L LbL samples at all deposition temperatures with the ATR-FTIR spectrum of Cu-BDC bulk powder as well as Cu-BDC films formed via spray deposition (Figure S9).^23^ In the IR spectrum of a 12 L sample spray-deposited at 20 °C, the carboxylate stretches are of approximately equal intensity and lack the strong signal enhancement seen in the 12 L LbL samples. Bulk Cu-BDC also shows no preferred orientation (*F* = 0.57, where a value of 0.5 would represent the totally isotropic orientation).

In addition to the preferred orientation, the IRRAS data show a significant evolution in the symmetric carboxylate stretch as both the number of deposition cycles and the deposition temperature increase (Figure 4). At 0 °C and all cycles, ν_s_(COO^–^) has a strong peak at 1404 cm^–1^ with a weak shoulder at approximately 1430 cm^–1^, based on fitting to the sum of the two Lorentzian peaks. Carboxylate peak positions are summarized in Table 1, along with the ring stretch mode ν(C=C). This peak fitting approach was applied to each spectral region of interest in Figure 4, and the details are provided in the Supporting Information with all spectral fits given in Figures S10–S13. For samples prepared at ambient temperature, the main ν_s_(COO^–^) feature (peak 1) is centered at around 1405 cm^–1^, but the intensity of the high-frequency shoulder at 1430 cm^–1^ (peak 2) becomes more prominent for the 12 L deposition cycles. At elevated temperature, the intensity of peak 1 at 1408 cm^–1^ decreases as peak 2 grows in with increasing deposition cycles, so that peak 2 is the most intense band at 1439 cm^–1^ after 12 deposition cycles. Two trends can be identified here. First, both the lower frequency ν_s_(COO^–^) band, peak 1, and the higher frequency ν_s_(COO^–^) shoulder or band, peak 2, show a slight blue shift with increasing temperature. Second, the prominence of peak 2 strongly depends on deposition temperature, as well as the number of deposition cycles. The appearance and growth of this feature accompanies the formation of fused, segmented nanorods observed by AFM.

**Figure 5:**
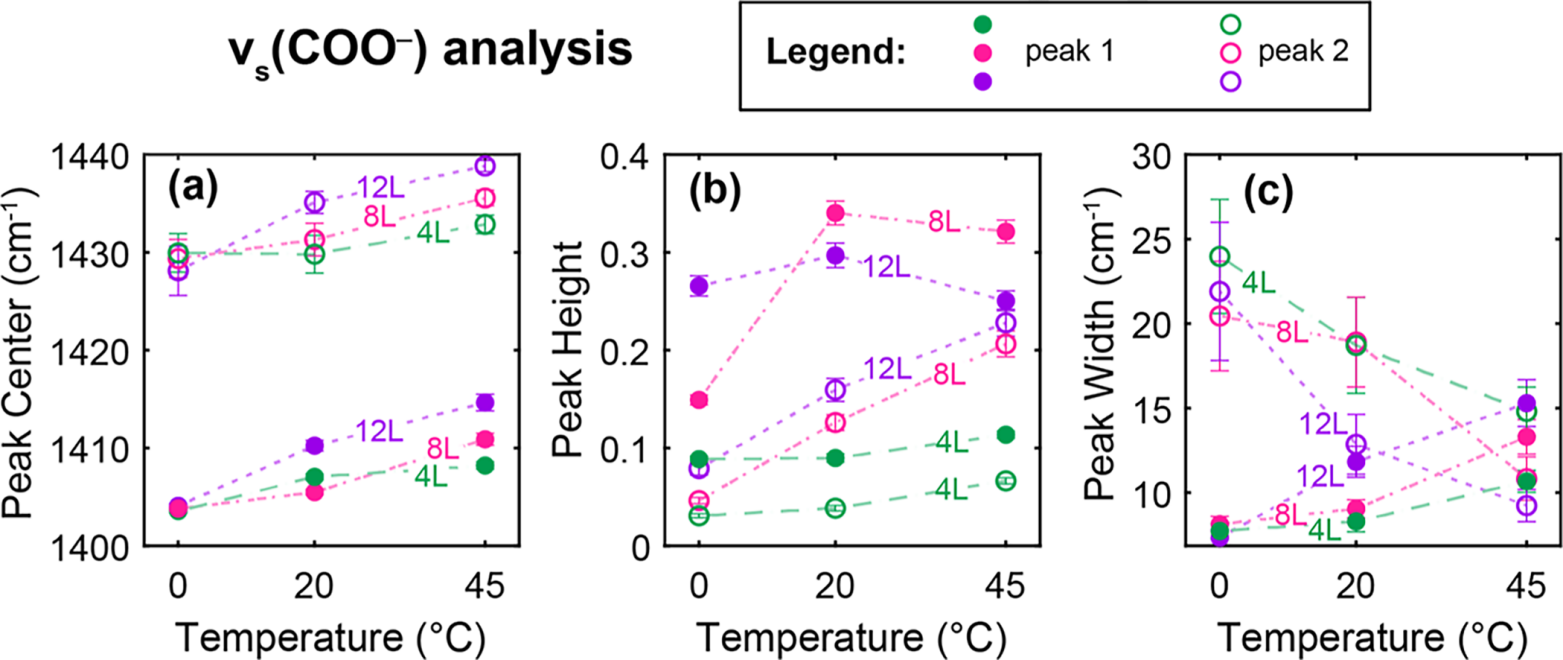
(a) Peak center, (b) height, and (c) width from fitting IRRA data of 4 L (green), 8 L (pink), and 12 L (purple) films as a function of temperature for the symmetric carboxylate stretch ν_s_(COO^–^). Closed circles denote the lower frequency peak in each region (peak 1), with open circles for the higher frequency peak that increases in intensity with temperature (peak 2). Error bars represent the standard error of the fit.

To more clearly identify the trends in the split bands for ν_s_(COO^–^) as a function of the deposition cycle and temperature (Figure 4a), the peak positions, heights, and widths from the spectral fit of the carboxylate region are shown as a function of deposition temperature in Figure 5a–c. Peak 1 represents the lower frequency peak (present at all deposition temperatures and cycles), and peak 2 is the higher frequency shoulder that grows with increasing temperature and number of cycles. In Figure 5a, both peaks’ positions shift to higher frequency as the deposition temperature increases from 0 to 45 °C. Figure 5b shows that peak 1 is more intense compared to peak 2 at all temperatures and deposition cycles, but peak 2 grows linearly with increasing deposition temperature. The growth of peak 2 occurs at the expense of peak 1 for 12 and 8 L, such that at 45 °C, the intensities are similar. Finally, from the peak widths in Figure 5c, peak 1 is very narrow at 0 °C but broadens with increasing deposition temperature, whereas peak 2 starts much broader but narrows as it grows in with increasing temperature. This effect is accelerated as the number of deposition cycles increases from 4 to 12.

The difference between the antisymmetric and symmetric carboxylate stretching frequencies, the separation value (Δ), is sensitive to the metal-carboxylate coordination mode.^40^ In MOFs with BDC linkers, coordination typically is described by a bridging bidentate configuration with values of Δ between 160 and 200 cm^–1^.^41,53^ As coordination goes from bridging to chelating, the value of Δ is reduced, in part because the O–C–O bond angle becomes smaller.^54^ The position of ν_as_(COO^–^) is relatively stable across the set of samples; the value of Δ at each temperature and deposition cycle for both peaks 1 (Δν(COO^–^)_1_) and 2 (Δν(COO^–^)_2_) are calculated in Table 1. All of the values suggest a bridging motif for the BDC linker, as expected. However, peak 2 shows a reduction in Δ by ∼20–30 cm^–1^. Comparing the average values across all layers for each temperature, Δν(COO^–^)_1_ is approximately 180 cm^–1^, while Δν(COO^–^)_2_ is around 155 cm^–1^. As a function of temperature, the average values of Δν(COO^–^)_1_ and Δν(COO^–^)_2_ are 182 ± 4 and 156 ± 3 cm^–1^ at 0 °C; 181 ± 2 and 157 ± 3 cm^–1^ at 20 °C; and 175 ± 3 and 151 ± 3 cm^–1^ at 45 °C, respectively. At each deposition temperature, there is a small reduction (3–5 cm^–1^) in both Δ values as the number of deposition cycles increases: the average Δν(COO^–^)_1_ and Δν(COO^–^)_2_ values are 181 ± 3 and 156 ± 4 cm^–1^ at 4 L; 181 ± 4 and 156 ± 4 cm^–1^ at 8 L; and 176 ± 4 and 152 ± 4 cm^–1^ at 12 L, respectively. This suggests that the segmented morphology, which becomes more pronounced at higher temperatures and higher surface coverage as shown in Figure 1, is associated with a reduction in Δ. While BDC remains in a bridging configuration, the emergent binding motif characterized by peak 2 suggests that the O–C–O bond angle may be reduced compared to the binding motif of peak 1 and that this configuration becomes more prominent at higher surface density and higher deposition temperature. Peak fitting parameters for ν_s_(COO^–^) are plotted as a function of the deposition cycle in Figure S14.

Band splitting is also observed for BDC deformation modes δ(CH)_18a_ and γ(CH)_11_in Figure 4b,c, particularly noticeable in the 12 L spectra and at higher deposition temperatures. The splitting of both these BDC C–H deformations leads to a lower frequency peak, in contrast to peak 2 for ν_s_(COO^–^). At 0 °C, δ(CH)_18a_ appears at 1027 cm^–1^. At 20 °C, this peak has a shoulder at 1019 cm^–1^ that becomes the most prominent in the 12 L sample. At 45 °C, this deformation mode is split with two peaks of roughly equal intensity, in which the lower frequency peak grows as the number of deposition cycles increases. A similar trend is seen for γ(CH)_11_, where at low-temperature deposition, a peak is seen at 837 cm^–1^, and at a higher temperature, a secondary peak grows in at 831 cm^–1^. The peak fitting parameters for all noncarboxylate modes are plotted as a function of deposition temperature in Figure S15.

A previous study of CO_2_ adsorption–desorption cycles demonstrated that BDC δ(CH)_18a_ was sensitive to the MOF structure’s compression and expansion, where a higher frequency peak appeared at high gas pressures when CO_2_ occupied the pores (expansion).^55^ These pore-sensitive IR breathing modes have since been commonly used as diagnostics of whether BDC-containing MOFs, such as MIL-47 or MIL-53,^55−57^ are in a “large pore” or “narrow pore” structure. When both the expanded and strained structures are coexistent, the ν_18a_ vibration is observed at both 1026 and 1018 cm^–1^, respectively.^57^ While the present films’ surMOF structure is distinct from these MOF powder structures, they too have BDC linkers that bridge metal centers. The nanorod structures seen by AFM are associated with the higher-frequency 1026 cm^–1^ feature (Figure 4b), whereas the platelets of nanorods accompany the growth of the lower-frequency peak at 1019 cm^–1^ (Figure 4c). The appearance of a lower-frequency peak in the other C–H deformation mode is similar. This observed trend may indicate a deformation-induced strain of the surMOF structure that accompanies the fusion of rods into nanoplatelets. The coexistence of these two bands can be understood by examining Figure 1f–i, which correspond to the 20 °C 12 L film and the 45 °C films for 4–12 L, where the nanostructures become elongated along one axis and clearly still possess distinct nanorods.

Band splitting is also observed for the sharp ν(OH) band at 3570 cm^–1^ as the layer number increases for deposition at ambient and elevated temperatures. In Figure 4d, the peak at 3570 cm^–1^ broadens slightly, and a higher frequency shoulder grows at the expense of the main peak. For fitting this spectral region, one peak was sufficient for all samples at 0 °C, but two peaks were necessary at higher temperatures. As seen in Figure S15, the higher-frequency peak of ν(OH) (peak 2) grows in and blue-shifts with an increase in deposition temperature at all deposition cycles. Similar to the observed trend in ν_s_(COO^–^), the intensity of peak 2 grows at the expense of lower-frequency peak 1 for ν(OH). Peak 1 does not broaden significantly as a function of deposition temperature, and peak 2 actually becomes narrower than peak 1 at the most elevated deposition temperature. Both features for ν(OH) remain relatively sharp, which is not consistent with hydrogen bonding or adsorption of the bound solvent or water. In that case, significant broadening and a red shift would be expected.^48,58−60^ Any residual H-bonding features are minor and not correlated to the spectral changes observed (Figure S7). Instead, the emergent band at 3611 cm^–1^ is blue-shifted by ∼40 cm^–1^ relative to the initial band (peak 1) with a similar bandwidth. This is reminiscent of the magnitude of spectral shifts when new binding motifs are created, as seen in mixed-metal MOFs,^61,62^ mixed-linker MOFs,^39,63^ or chemical transformation of bulk MOFs into layered structures.^43,64^ Notably, splitting in ν(OH) was observed to be correlated with pore breathing modes in MIL-53 structures,^57^ with the higher-frequency peak assigned to the “narrow pore” (or more strained) structure. This is consistent with the observed IRRAS data for the Cu-BDC surMOF films, where segmented nanoplatelet formation is accompanied by the growth of both the lower-frequency C–H deformation peak and the higher-frequency O–H stretch, both of which suggest that segmentation induces strain in one direction as the nanorods fuse.

Overall, as both deposition temperature and number of deposition cycles increase, new peaks are observed that suggest structural change that affects the linker binding, as in the blue-shifted splitting of the symmetric carboxylate stretch, and that is associated with a narrower, or more collapsed, BDC linker framework, as in the red-shifted splitting of the aromatic C–H deformation modes and blue-shifted splitting of the hydroxyl stretch. The IR data match with the morphologies observed by AFM. At 20 °C deposition temperature, sections of nanorods begin to align for 8 L (Figure 1e), which becomes more significant at 12 L (Figure 1f), and the IR data show intermediate or weak shoulders for split bands. At elevated temperature, significant alignment of the nanorods within the platelet structure occurs at all surface densities (Figure 1g–i), and for all cases, split bands are observed in IR for each of the reporter modes ν_s_(COO^–^), δ(CH), γ(CH), and ν(OH) in Figure 4. This trend becomes more pronounced at higher deposition cycles.

The direction of splitting for each peak can be indicative of strain induced in one direction as nanoplatelets are formed through the fusing of segmented nanorods. The reduction in Δ may represent a smaller or more strained O–C–O bond angle, similar to what is seen as bridging carboxylate binding becomes chelating. Here, the binding remains bridging, but nanoplatelets composed of nanorod segments are hypothesized to have stress at nanorod fusion sites that causes these spectral changes, such that both less strained and more strained IR features are observed at higher deposition cycles and at a higher deposition temperature.

## Conclusions

Deposition temperature (0, 20, and 45 °C) influences the thin film formation of Cu-BDC surMOF throughout a series of deposition cycles (4, 8, and 12) as demonstrated by characterization with AFM, ellipsometry, XRD, and IRRAS. Increasing the number of deposition cycles and temperature resulted in more film deposition. For all temperatures and number of cycles, XRD shows that Cu-BDC thin films are crystalline, and AFM images reveal thin film formation of isolated nanostructures. For films formed at 0 and 20 °C, the crystallites observed by AFM are similar nanorod-like structures attached to the substrate with fewer and shorter crystallites observed at 0 °C. However, for the crystallites observed for films deposited at 45 °C, vertical segmented nanoplatelets were observed, appearing to be fused nanorods aligned in one direction. All characterization techniques showed that more film deposition occurred with an increasing number of deposition cycles and that films deposited at higher temperatures had increased film thickness and coverage.

IRRAS characterized the chemical binding within these surMOF nanocrystallites, confirming preferred orientation within these surface-bound nanorod and nanoplatelet structures as shown by the enhanced symmetric carboxylate stretch and the absence of the corresponding antisymmetric stretch. From surface selection rules for anisotropic films on gold, this indicates that the Cu–Cu axis of the paddlewheel structure within the nanocrystallites is oriented parallel to the substrate. At elevated temperatures and with an increasing number of deposition cycles, a secondary higher frequency peak (∼1435 cm^–1^) was observed to increase for this symmetric carboxylate stretch. As this peak grows in, the preferred orientation is maintained, but the higher frequency of the new carboxylate peak suggests the emergence of a more strained binding motif that corresponds to the formation of platelet-like structures and high-density coverage of nanostructures observed by AFM. Additional trends were observed in the splitting of BDC CH deformation modes as well as the hydroxyl stretch, which have previously been identified as modes sensitive to pore deformation and compression in related MOF structures. This further supports the presence of a strained configuration along one direction that accompanies the fused nanorod segments of the nanoplatelets.

## Supporting Information Available

Additional AFM images and associated line scans; linear fits for AFM roughness data and ellipsometric thickness; annotated IRRA spectra (400–4000 cm^–1^); expanded hydroxyl region of IRRA spectra; integrated areas of IRRA spectra as a function of deposition cycle and temperature; comparison of IRRAS data for LbL immersion vs LbL spray samples and ATR spectrum for bulk powder; peak fitting procedure for IRRAS data; peak fits for symmetric carboxylate stretch, ring deformation modes [δ(CH) and γ(CH)], and hydroxyl stretch; peak fit parameters (center, height, and width) for CH deformation modes and hydroxyl stretch as a function of deposition temperature; and peak fit parameters for the symmetric carboxylate stretch as a function of deposition cycle (PDF)
