# Supplementary material for: Influence of Deposition Temperature on Cu-BDC Surface-Anchored Metal–Organic Framework Formation
Source: J Phys Chem C Nanomater Interfaces. 2024 Dec 19;129(1):732–43. doi: 10.1021/acs.jpcc.4c06638 (PMC11726682; doi:10.1021/acs.jpcc.4c06638)
Supplement: Supplementary file 1 — jp4c06638_si_001.pdf [file jp4c06638_si_001.pdf]

Supporting Information: **Influence of Deposition Temperature on Cu-BDC Surface-Anchored Metal-Organic Framework Formation**

Authors:
Skylar J. Delozier,† Dayton L. Maglich,† Katherine E. Coffin,‡ Katherine S. Euston,† Catherine M. Mauck,‡ Mary E. Anderson†*
† Furman University, Greenville, South Carolina 29613, United States
‡ Kenyon College, Gambier, Ohio 43022, United States
*Corresponding author: maryelizabeth.anderson@furman.edu

Included:

Figure S1. AFM images (2.5 μm x 2.5 μm) of Cu-BDC on MHDA-functionalized substrates associated with Figure 1 and 2 in the manuscript. (Page S2)

Figure S2. Representative AFM image (1 μm x 1 μm) and three-dimensional rendering. (Page S3)

Figure S3. Three-dimensional images (2.5 μm x 2.5 μm) of Cu-BDC surMOFs with x:y:z ratio of 1:1:1 for comparison to Figure 2 in manuscript. (Page S4)

Figure S4. Line scans associated with images in Figure S1. (Page S5)

Figure S5. Linear fits for a) AFM roughness data and b) ellipsometric thickness data. (Page S6)

Figure S6. Thin film IRRA spectra from 400 – 4000 cm^{-1} with annotated vibrational modes. (Page S7)

Figure S7. Expanded hydroxyl region thin film IRRA spectra normalized to MHDA peaks. (Page S8)

Figure S8. Integrated area of IRRAS as a function of deposition cycle and temperature. (Page S8)

Figure S9. Comparison of IRRAS data for surMOF samples fabricated by LbL solution-phase vs. spray deposition methods alongside ATR data for bulk powder. (Page S9)

Description of peak fitting procedure for IRRAS data. (Page S10)

Figure S10. Peak fits of symmetric carboxylate stretch ν_s(COO). (Page S11)

Figure S11. Peak fits of ring deformation mode δ(CH). (Page S11)

Figure S12. Peak fits of ring deformation mode γ(CH). (Page S12)

Figure S13. Peak fits of hydroxyl stretch ν(OH). (Page S12)

Figure S14. Peak fit parameters (center, height, width) as a function of deposition cycle for $\nu_s(COO^-)$. (Page S13)

Figure S15. Peak fit parameters (center, height, width) for CH deformation modes and hydroxyl stretch as a function of deposition temperature. (Page S14)

References (Page S15)

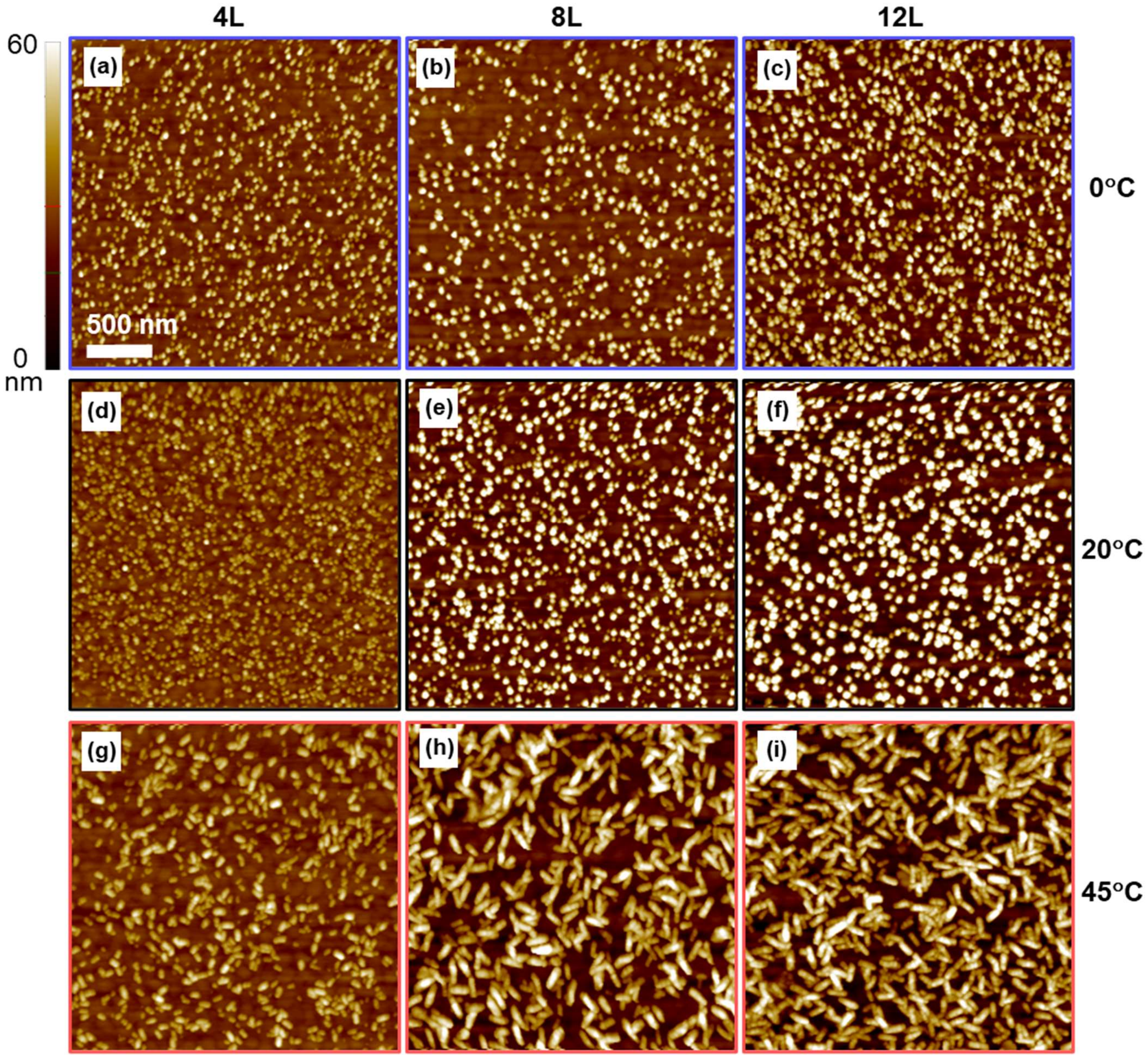

Figure S1. AFM images (2.5 μm x 2.5 μm) of Cu-BDC on MHDA-functionalized substrates collected at 4, 8, and 12 deposition cycles (L) at (a-c) lowered, (d-f) ambient, and (g-i) elevated temperatures. These images are associated with Figure 1 and 2 in the manuscript.

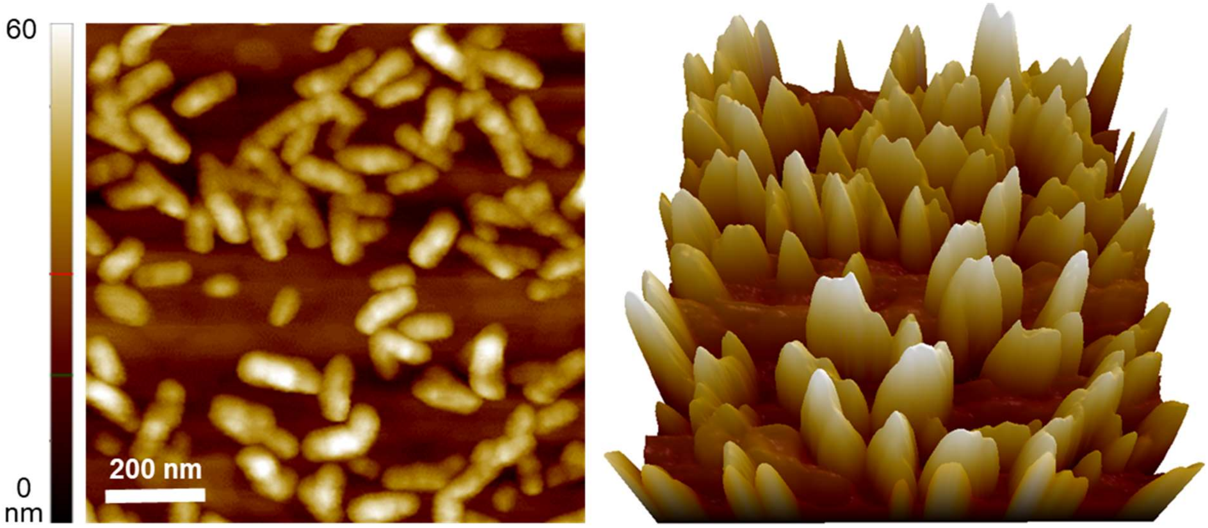

Figure S2. Representative AFM image (1 μm x 1 μm) and three-dimensional rendering of Cu-BDC on MHDA-functionalized substrate collected after 8 deposition cycles at elevated temperature.

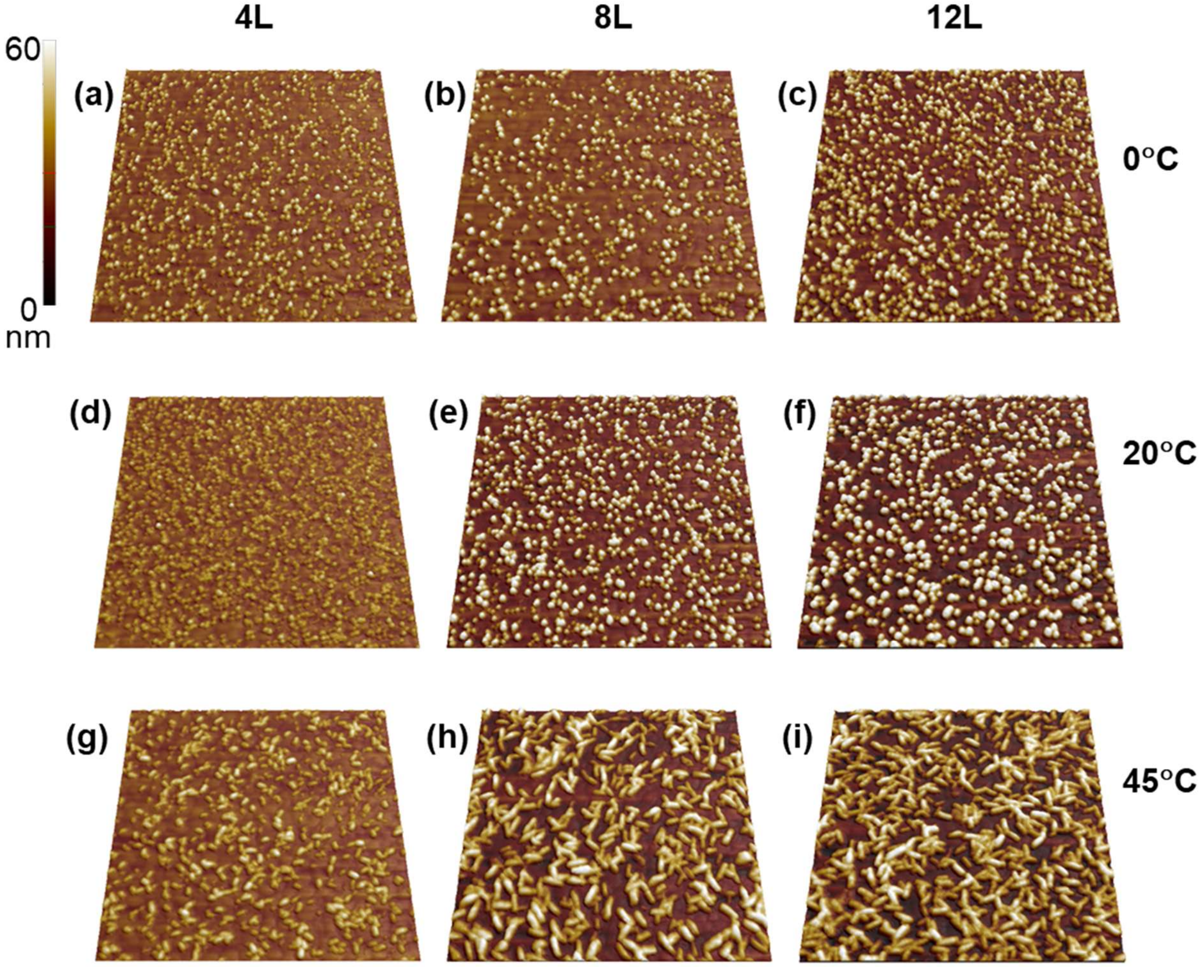

Figure S3. Three-dimensional images (2.5 μm x 2.5 μm) of Cu-BDC surMOFs on carboxylic acid-functionalized substrates. These perspectives were rendered from AFM images to convey the structure and density of the nucleated crystals formed throughout the deposition process after 4,8, and 12 deposition cycles (L) at varying precursor temperatures. These are the same images as those shown in Figure 2 of the manuscript, however here the x:y:z ratio is 1:1:1 while in the manuscript the ratio is 1:1:8 to emphasize the particle shape.

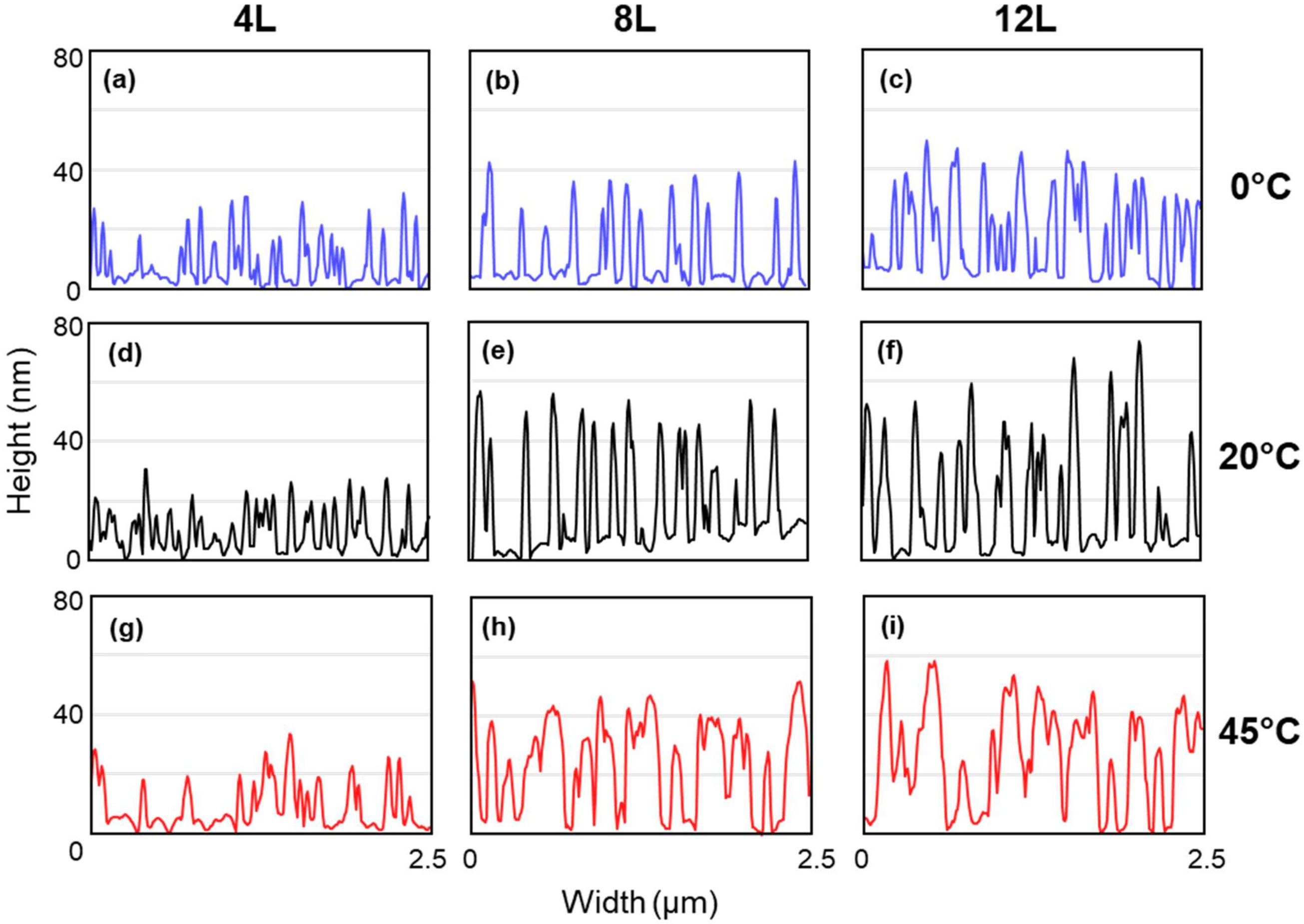

Figure S4. Representative line scans from AFM images in Figure S1. Line scans are arranged in 4, 8, and 12 deposition cycles (L) for samples deposited at (a-c) lowered, (d-f) ambient, and (g-i) elevated temperatures of 0, 20, and 45 °C, respectively.

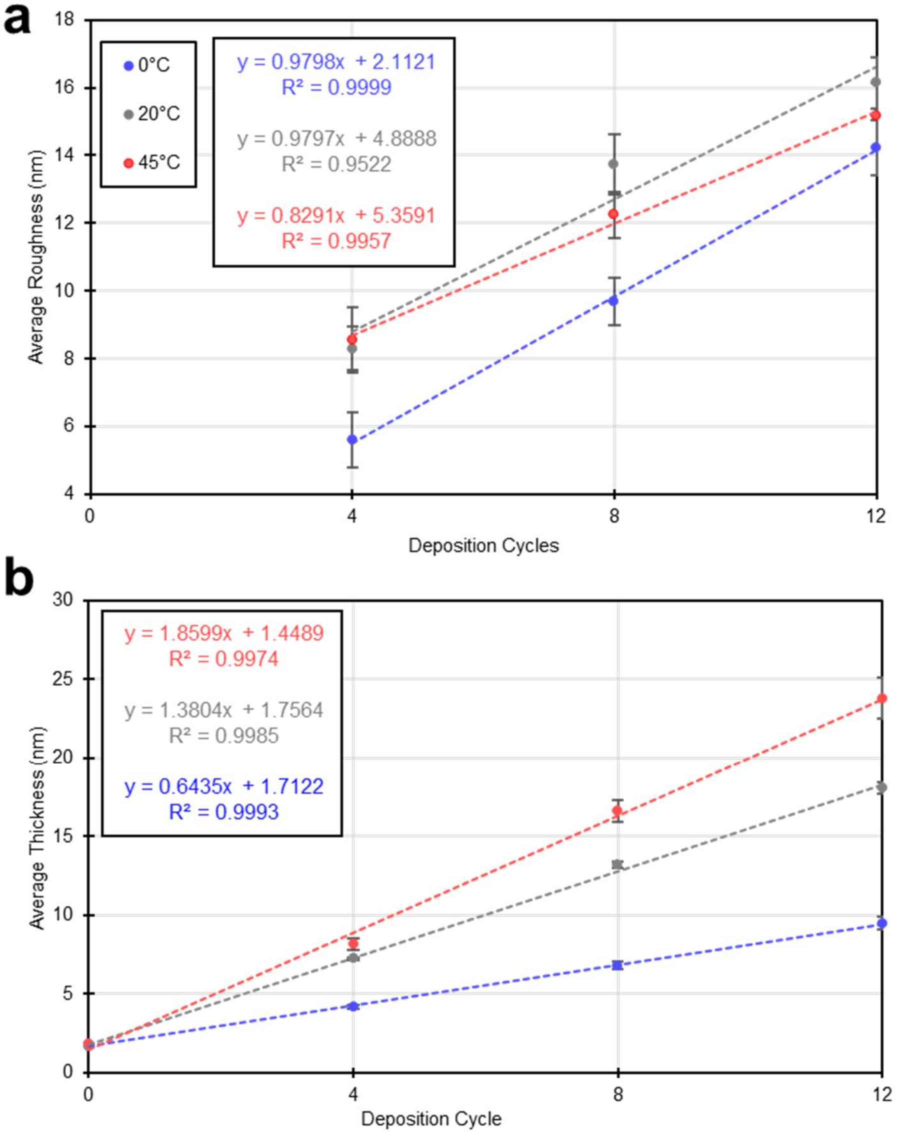

Figure S5. (a) Average roughness values collected from atomic force microscopy imaging (2.5 µm x 2.5 µm) and (b) average ellipsometry thicknesses with standard deviations. Both plots include linear fits of samples collected at 0, 20, and 45 °C after 4, 8, and 12 deposition cycles (L).

Ellipsometry is a key instrument routinely utilized in this study to demonstrate the uniformity of the film across a single sample as well as the reproducibility amongst multiple fabricated samples. Note that ellipsometry data provides an optical thickness for the film based on the index of refraction for the film (n_f) value of 1.5 and extinction coefficient for the film (k_f) value of 0, as well as the measured index of refraction and extinction coefficient for each substrate. The discontinuous and rough nature of the films in this study is not considered to determine the optical thickness within the data fitting model in the Gaertner ellipsometer measurement program (LGEMP).

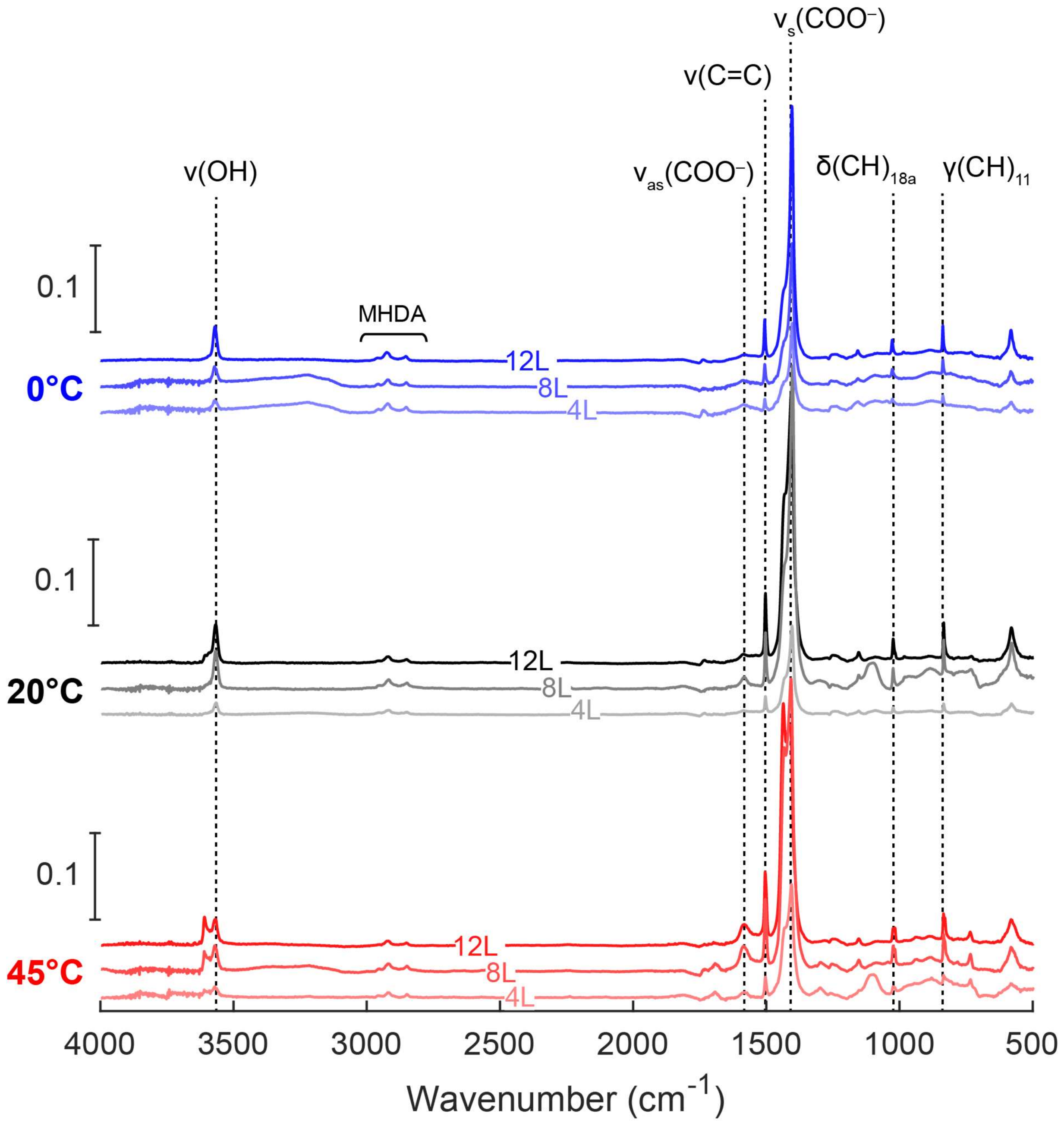

Figure S6. Thin film spectra from IRRAS measurements, for deposition cycles 4 L, 8 L, and 12 L, at temperatures 0 °C (blue), 20 °C (black), and 45 °C (red). Spectra are offset for clarity. Scale bar represents 0.1 absorbance units. Dashed lines serve as guides to the eye for vibrational modes discussed in main text. Methylene stretches associated with SAM layer of 16-mercaptohexadecanoic acid (MHDA) are labeled.

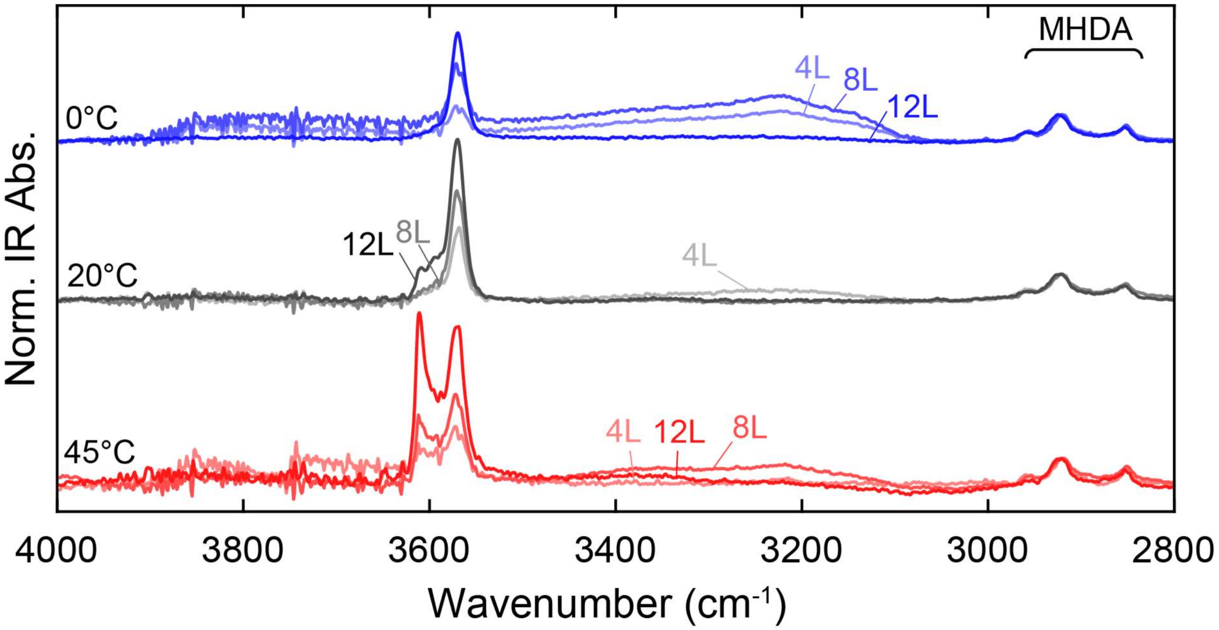

Figure S7. Thin film spectra from IRRAS measurements with expanded hydroxyl region, for deposition cycles 4 L, 8 L, and 12 L at temperatures 0 °C (blue), 20 °C (black), and 45 °C (red), normalized to the SAM vibrational modes (MHDA) below 3000 cm^{-1}.

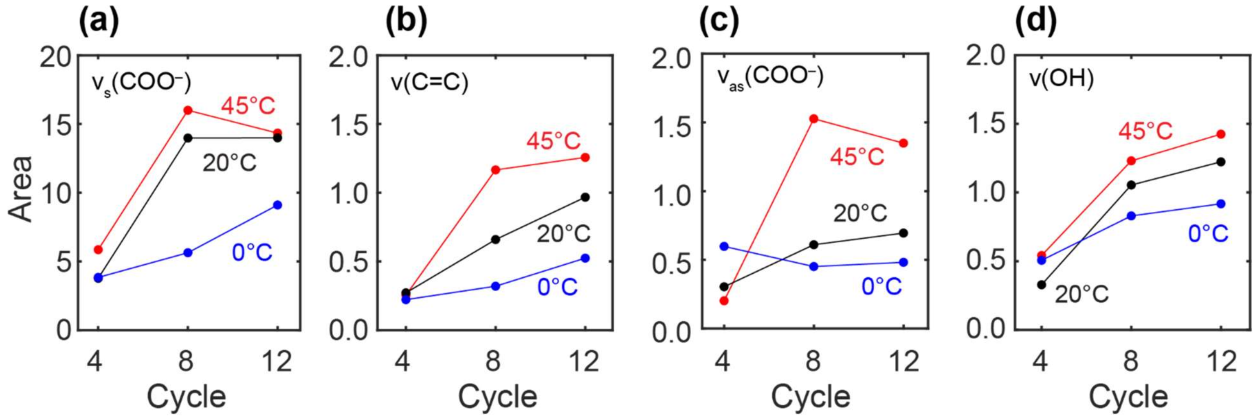

Figure S8. Integrated area under spectral regions in IRRAS of thin films as a function of deposition cycle and temperature for (a) $\nu_s(COO^-)$, 1325–1482 cm^{-1}; (b) ν(C=C), 1490–1515 cm^{-1}; (c) $\nu_{as}(COO^-)$, 1536–1630 cm^{-1}; and (d) ν(OH), 3540–3622 cm^{-1}.

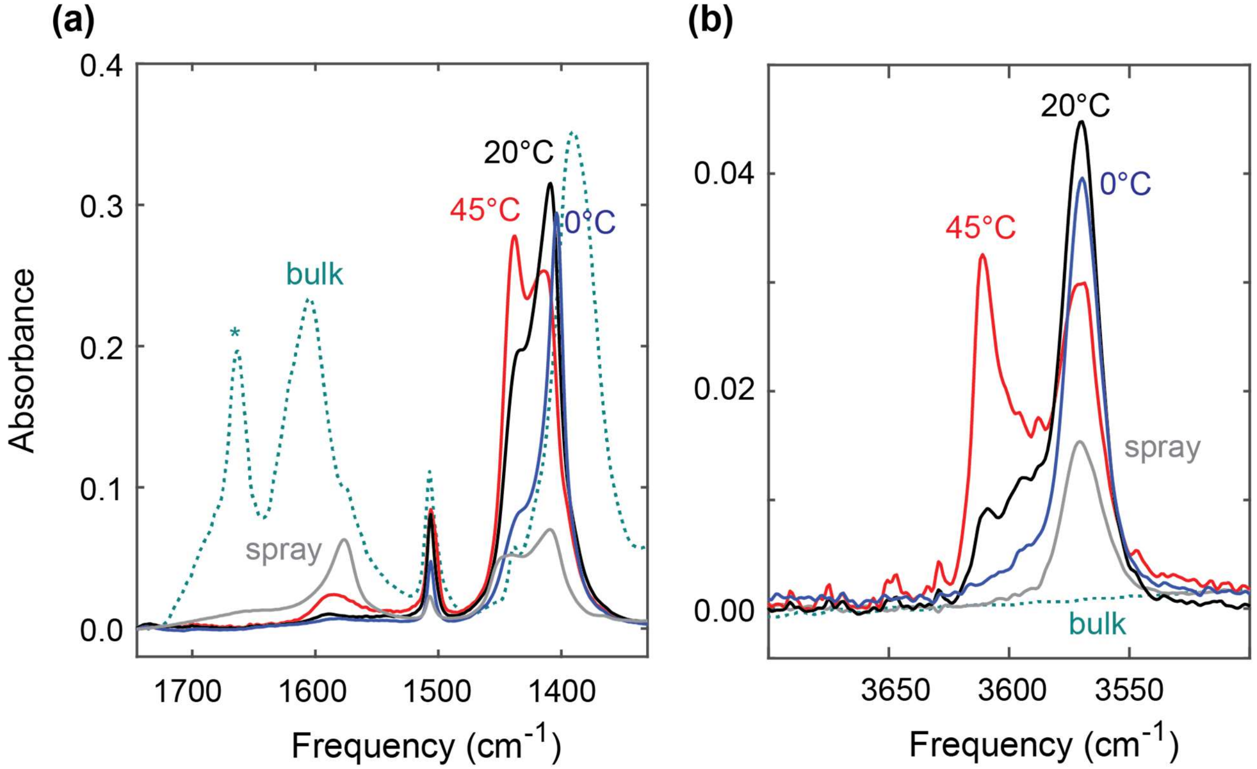

Figure S9. IRRAS data of (a) carboxylate region and (b) hydroxyl region for 12 L layer-by-layer (LbL) solution-phase deposition samples at 0 °C (blue), room temperature (black), and 45 °C (red), compared to room temperature LbL spray-deposited sample (grey)[1] and bulk powder spectrum acquired by ATR (teal, dotted line). Asterisk denotes dimethyl formamide consistent with the synthesis of bulk powder.[2]

Description of peak fitting procedure for IRRAS data.

The IRRAS spectra for films deposited at 0°C, 20°C, and 45°C and 4, 8, and 12 L deposition cycles were fit with a non-linear least squares algorithm to a sum of N Lorentzian peaks in each region with band splitting, in a custom script written in MATLAB 2024a.

For the carboxylate stretching region, the fit was performed from 1350–1650 cm^{-1} initialized with $N = 4$ peaks for $\nu_{as}(COO^{-})$, $\nu(C{=}C)$, and Peak 1 as well as Peak 2 of $\nu_{s}(COO^{-})$, where Peak 1 is present at all temperatures and Peak 2 is a higher frequency shoulder that increases in intensity with increasing deposition cycle and temperature.

For the C-H deformation modes δ(CH) and γ(CH), the fit was performed from 1000–1050 cm^{-1} and 815–850 cm^{-1} respectively. The weaker signal in both regions required a linear background subtraction to be applied prior to fitting. Overall the C-H deformation modes are characterized by lower signal-to-noise compared to the other spectral regions of interest, and the error of the fits were much greater. Both fits were initialized with $N = 2$ peaks for Peak 1 and Peak 2, where higher frequency Peak 1 is present at all temperatures and Peak 2 is a lower frequency shoulder that increases in intensity with increasing deposition cycle and temperature.

For the hydroxyl stretch region the fit was performed from 3500–3700 cm^{-1}. This spectral region suffered from low signal-to-noise at the lowest number of deposition cycles. At 0 °C, one peak was used to fit the spectra. Above this temperature, $N = 2$ peaks were used for Peak 1 and Peak 2, where the lower frequency Peak 1 is present at all temperatures and Peak 2 is a higher frequency shoulder that grows in with increasing deposition cycle and temperature.

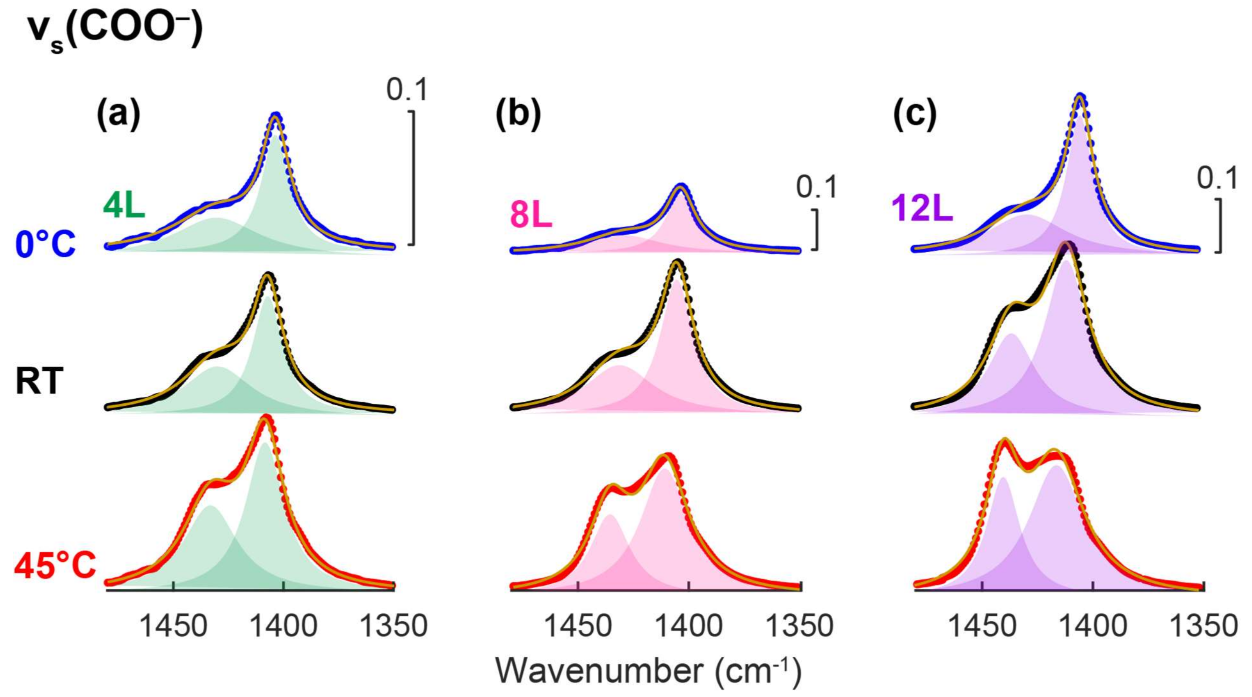

Figure S10. Fit of symmetric carboxylate stretch $\nu_s(COO^-)$ for films deposited at 0 °C (top, blue), room temperature (middle, black), and 45 °C (bottom, red). Spectral region was fit to the sum of two Lorentzian peaks (yellow line) for (a) 4 L (green), (b) 8 L (pink), and (c) 12 L (purple) samples. Scale bars for (a)-(c) represent 0.1 absorbance units.

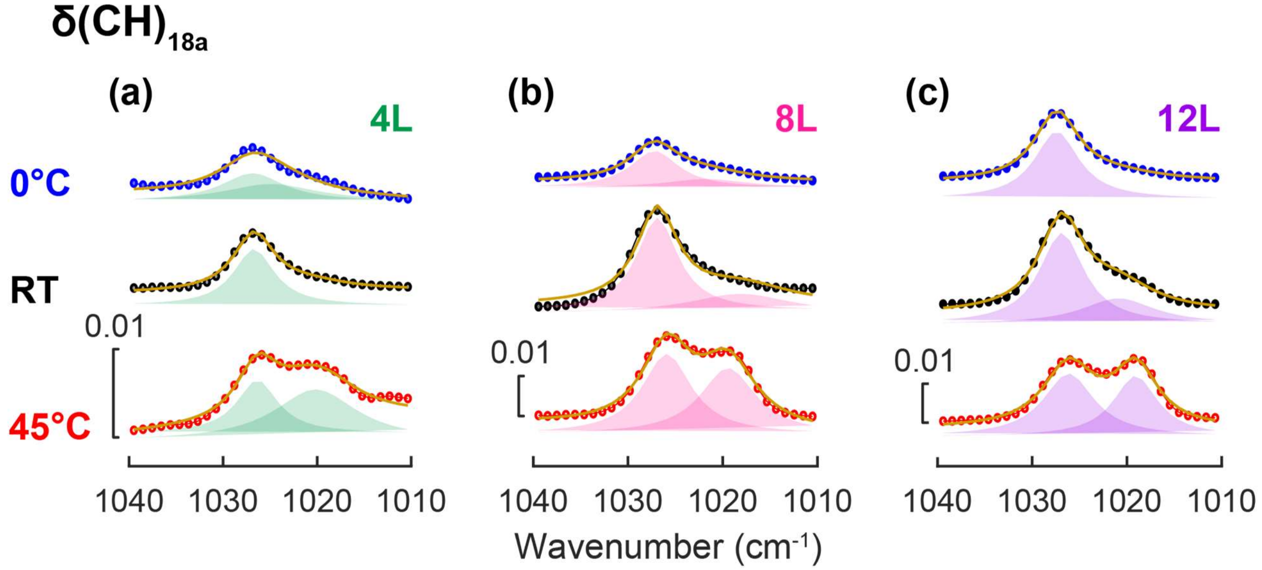

Figure S11. Fit of CH deformation mode δ(CH) for films deposited at 0 °C (top, blue), room temperature (middle, black), and 45 °C (bottom, red). A linear background subtraction was applied to the spectral region prior to fitting to the sum of two Lorentzian peaks (yellow line) for (a) 4 L (green), (b) 8 L (pink), and (c) 12 L (purple) samples, with the exception of 4 L RT and 12 L 0°C which were fit to a single Lorentzian peak. Scale bars for (a)-(c) represent 0.01 absorbance units.

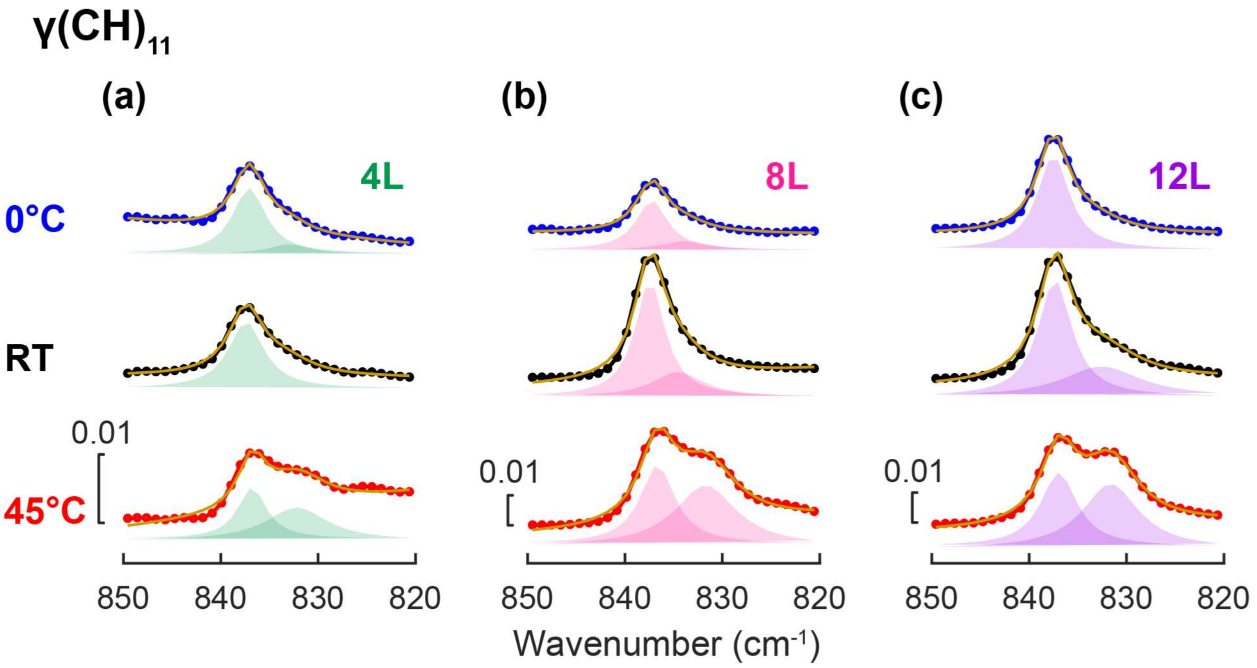

Figure S12. Fit of CH deformation mode γ(CH) for films deposited at 0 °C (top, blue), room temperature (middle, black), and 45 °C (bottom, red). A linear background subtraction was applied to the spectral region prior to fitting to the sum of two Lorentzian peaks (yellow line) for (a) 4 L (green), (b) 8 L (pink), and (c) 12 L (purple) samples. Scale bars for (a)-(c) represent 0.01 absorbance units.

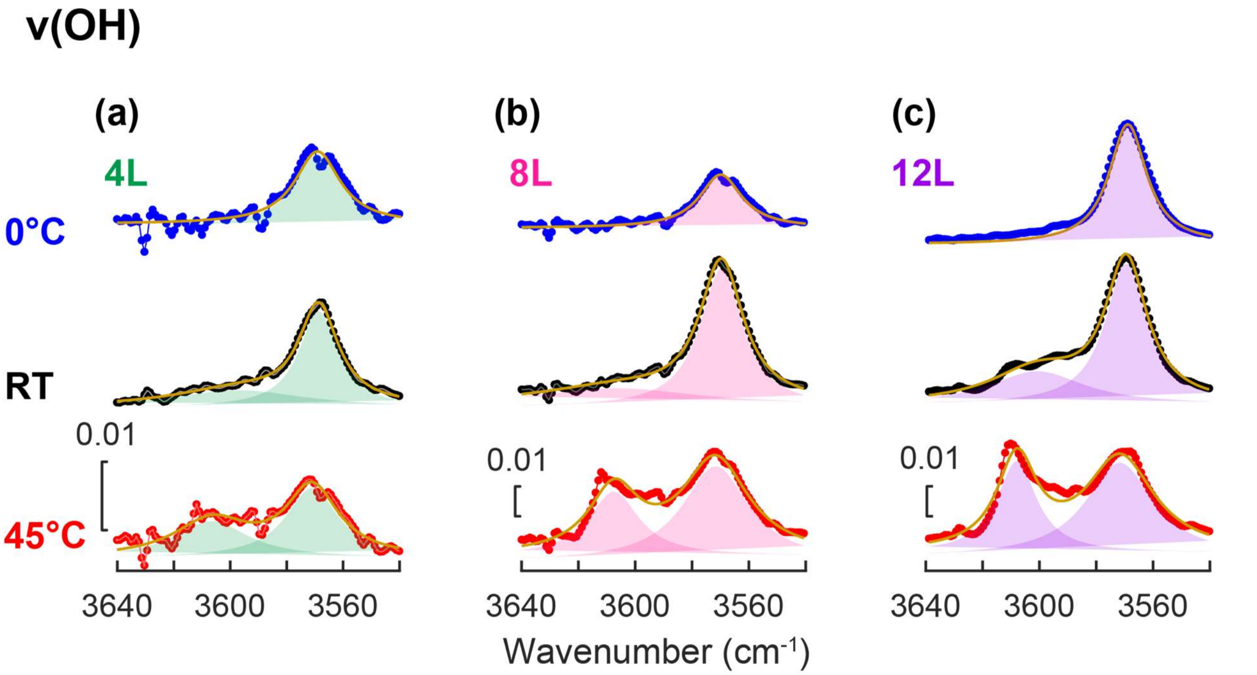

Figure S13. Fit of hydroxyl stretch ν(OH) for films deposited at 0 °C (top, blue), room temperature (middle, black), and 45 °C (bottom, red). Fit to spectral region is given (yellow line) for (a) 4 L (green) fit to a single Lorentzian peak, and for (b) 8 L (pink), and (c) 12 L (purple) samples fit to the sum of two Lorentzian peaks. Scale bars for (a)-(c) represent 0.01 absorbance units.

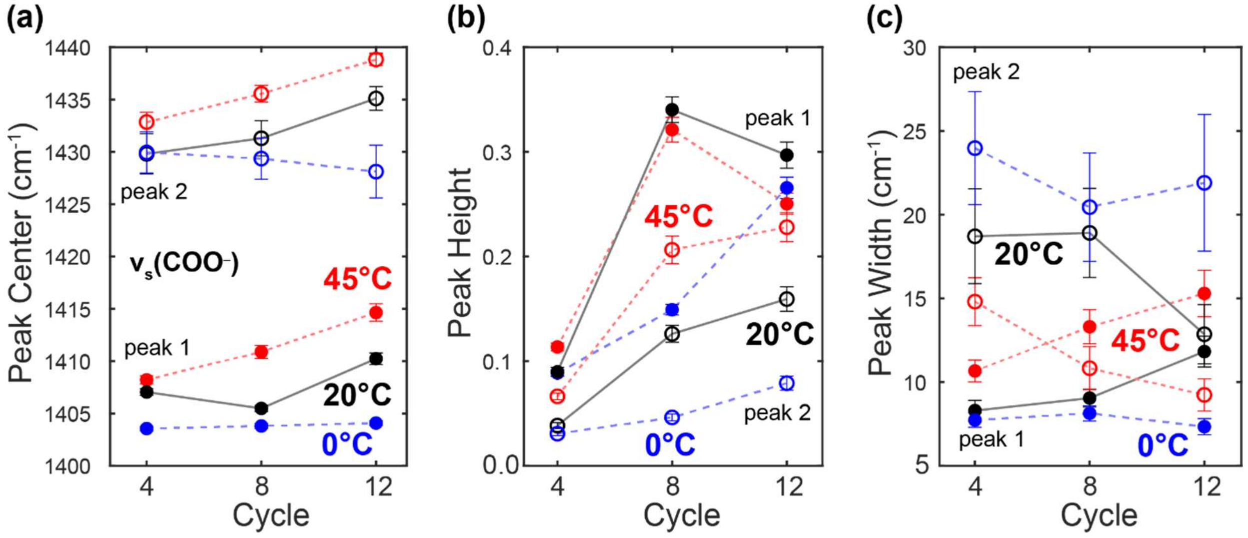

Figure S14. Peak fit parameters for symmetric carboxylate stretch $\nu_s(COO^-)$ of films deposited at 0 °C (blue), room temperature (black), and 45 °C (red) as a function of deposition cycles 4 L–12 L, where Peak 1 is the lower frequency peak (closed circles) and Peak 2 is the higher frequency peak (open circles). (a) Peak centers, (b) peak heights, and (c) peak widths. Error bars represent standard error of the fit.

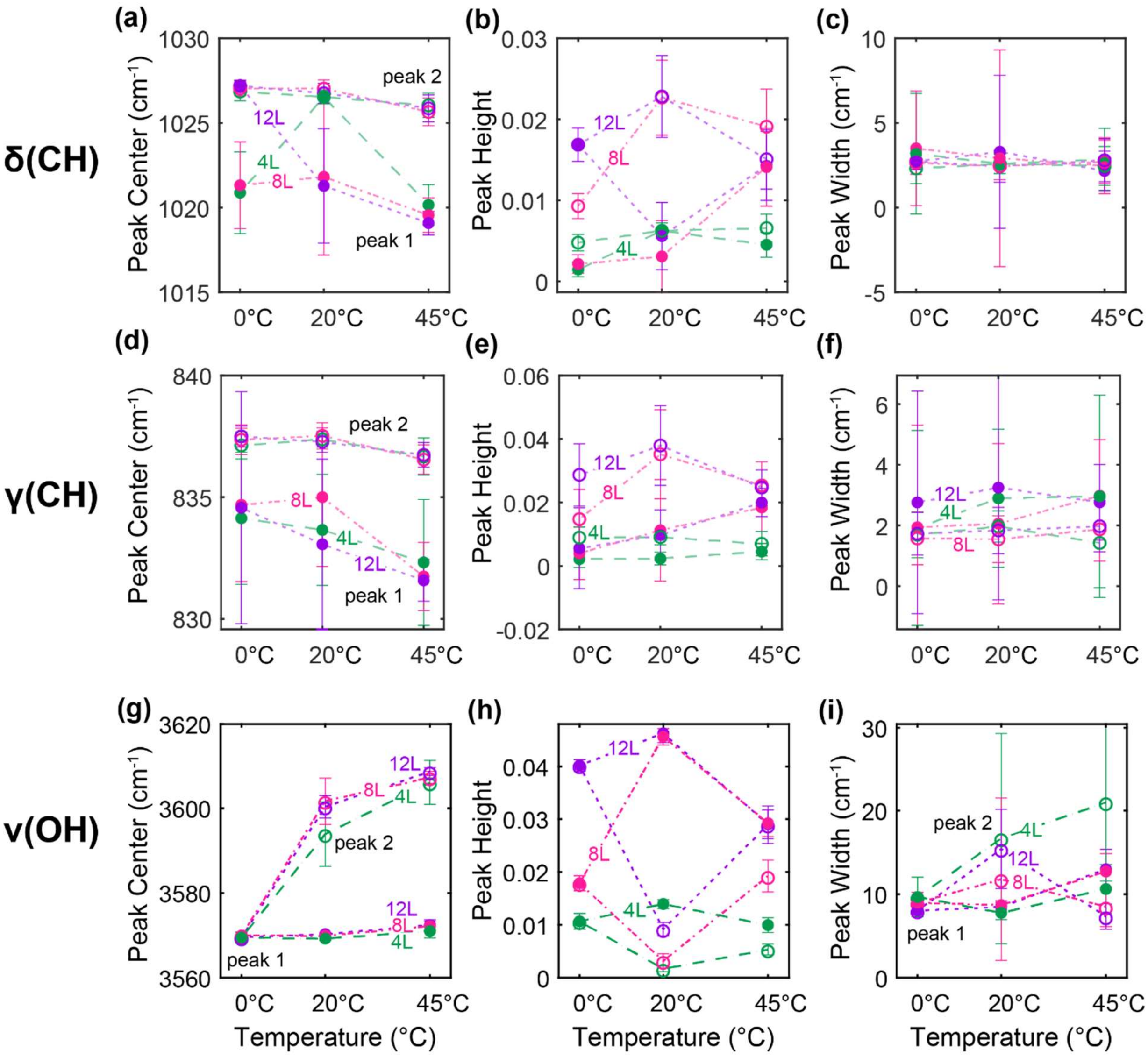

Figure S15. Peak center, height, and width from fitting IRRA spectra of 4 L (green), 8 L (pink), and 12 L (purple) films as a function of temperature for CH deformation modes (a)-(c) $\delta(CH)_{18a}$; (d)-(f) $\gamma(CH)_{11}$; and hydroxyl stretch (g)-(i) ν(OH). Closed circles denote the lower frequency peak in each region (peak 1), with open circles for the higher frequency peak that increases in intensity with temperature (peak 2). Error bars represent the standard error of the fit.

References:

(1) Dhanapala, B. D.; Maglich, D. L.; Anderson, M. E. Impact of Surface Functionalization and Deposition Method on Cu-BDC surMOF Formation, Morphology, Crystallinity, and Stability. *Langmuir* **2023**, *39* (34), 12196–12205. https://doi.org/10.1021/acs.langmuir.3c01505.

(2) Carson, C. G.; Hardcastle, K.; Schwartz, J.; Liu, X.; Hoffmann, C.; Gerhardt, R. A.; Tannenbaum, R. Synthesis and Structure Characterization of Copper Terephthalate Metal–Organic Frameworks. *Eur J Inorg Chem* **2009**, *2009* (16), 2338–2343. https://doi.org/10.1002/ejic.200801224.
